# Catalytic Role of Methanol in Anodic Coupling Reactions Involving Alcohol Trapping of Cation Radicals

**DOI:** 10.1021/acs.joc.4c02227

**Published:** 2024-12-03

**Authors:** Shahriar N. Khan, John H. Hymel, John P. Pederson, Jesse G. McDaniel

**Affiliations:** School of Chemistry and Biochemistry, Georgia Institute of Technology, Atlanta, Georgia 30332-0400, United States

## Abstract

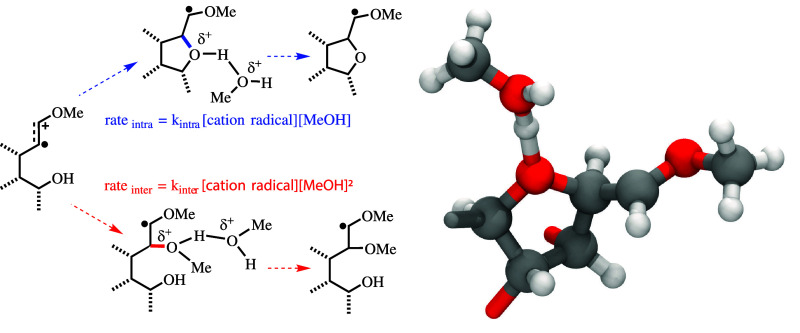

In anodic electrosynthesis, cation radicals are often key intermediates that can be highly susceptible to nucleophilic attack and/or deprotonation, with the selectivity of competing pathways dictating product yield. In this work, we computationally investigate the role of methanol in alcohol trapping of enol ether cation radicals for which substantial modulation of the reaction yield by the solvent environment was previously observed. Reaction free energies computed for intramolecular coupling unequivocally demonstrate that the key intramolecular alcohol attack on the oxidized enol ether group is catalyzed by methanol, proceeding through overall second-order kinetics. Methanol complexation with the formed oxonium ion group gives rise to a “Zundel-like”, shared proton conformation, providing a critical driving force for the intramolecular alcohol attack. Free energies computed for methanol solvent attack of enol ether cation radicals demonstrate an analogous mechanism and overall third-order kinetics, due to similar complexation from a secondary methanol molecule to form the “Zundel-like”, shared proton conformation. As catalyzed by methanol, both intramolecular alcohol attack and methanol attack on the oxidized enol ether group are barrierless or low-barrier reactions, with kinetic competition dictated by the conformational free energy profile of the cation radical substrate and the difference in reaction rate orders.

## 1 Introduction

Electrosynthesis is a powerful tool in synthetic organic chemistry, enabling polarity inversion (“umpolung”) of functional groups through oxidation/reduction under relatively mild reaction conditions.^1−10^ Anodic coupling reactions are an important class of electrosynthesis transformations, enabling carbon–carbon or heteroatom bond formation.^11^ Anodic coupling reactions have been employed for a variety of organic synthetic transformations,^1,9,12,13^ including synthesis of homo- and heterocyclic ring products,^9^ polycyclics,^14−18^ biaryls,^19,20^ natural product precursors,^21,22^ and total synthesis applications.^5,23,24^ In this work, we focus on *intramolecular* anodic coupling reactions, as pioneered by Moeller and coworkers,^1,9,12,13,25−31^ in which various target cyclization products can be synthesized from substrates containing electron-rich olefin initiators, such as enol ether groups. High yields and selectivities of intramolecular, anodic coupling reactions can be achieved through a strategic choice of functional groups and substituents. Because electrogenerated cation radicals are often the reactive intermediates, which may have very short, e.g., nanosecond lifetimes,^32−35^ electrolysis of a substrate may lead to a distribution of products with yield/selectivity depending sensitively on reaction conditions.^29^

With exception, methanol is often utilized as the solvent or cosolvent comprising the electolyte for such anodic, intramolecular coupling reactions.^9^ Practical reasons for this choice include that, being protic, methanol facilitates hydrogen evolution as the counter, cathodic half-reaction during the electrolysis, and additionally methanol participates in nucleophilic trapping of dication intermediates (e.g., forming an acetal) to yield the neutral product substrate. Beyond these practical considerations, however, in numerous cases, it has been empirically demonstrated that the methanol content of the solvent/electrolyte has a substantial influence on the overall selectivity and yield of the target anodic coupling reaction. For example, Xu and Moeller found that electrosynthesis of a tetramethoxyfuranose substrate via anodic intramolecular coupling reaction resulted in 85% yield in pure methanol solvent with LiClO_4_ electrolyte, while only 20–30% yield was achieved utilizing MeOH/THF cosolvent mixtures with Et_4_NOTs electrolyte.^29^ However, for similar anodic intramolecular coupling reactions forming tetrahydrofuran products, it was found that yield was optimized utilizing a cosolvent with reduced methanol content compared to pure methanol.^28^ As another example, Redden and Moeller found that for intramolecular anodic coupling of a bis-enol ether substrate in MeOH/CH_2_Cl_2_ cosolvent, switching from 0.1 M LiClO_4_ to 0.5 M Et_4_NOTs electrolyte improved product yield from 21% to 60%, presumably due to the greasy Et_4_NOTs electrolyte excluding MeOH from the electrical double-layer reaction zone.^30^ These experiments, as well as others,^9,13^ clearly indicate that methanol plays a crucial but complex role in dictating the selectivity/yield of certain anodic coupling reactions.

Empirically, it has been extensively demonstrated that methanol can trap cation radicals through nucleophilic attack.^11^ Certain methanol trapping pathways would be incompatible with the intended product formation, yielding side products instead. For anodic coupling reactions in methanol solvent or cosolvent, the target nucleophilic attack to the cation radical intermediate must thus compete with methanol attack. There are several possibilities for why and how an intended anodic coupling reaction could achieve high yield/selectivity within a methanol-rich solvent. First, the target nucleophile could simply be a stronger nucleophile than methanol, thus outcompeting methanol trapping of the cation radical intermediate. This can certainly explain the success of some such reactions, but in this regard it should be appreciated that intramolecular alcohol attack to cation radicals provides high-yield electrosynthesis products in many cases.^28^ A second explanation specific to intramolecular coupling reactions is that the intramolecular coupling may have a larger rate prefactor relative to methanol trapping, based on the proximity of the nucleophile to the cation radical. A final and more subtle explanation is that if nucleophilic attack to the cation radical occurs within the electrical double-layer region near the anode surface, methanol concentration could be dramatically reduced within the double layer, particularly if greasy electrolytes are utilized.^27,30^

Kinetic studies by Oyama et al. have provided important insights regarding the mechanism of alcohol (and similarly, water) trapping of cation radicals.^36,37^ Detailed kinetic analysis revealed that rates of methanol or water trapping of anthracene derivative cation radicals were first order in substrate concentration but *second order* in methanol (or water) concentration. This conclusion would seem surprising, as *a priori,* one would probably propose an overall second-order rate equation, being first order in substrate and first order in nucleophile concentration. Indeed, an overall second-order rate equation was previously utilized to fit rate constants for methanol and/or water trapping of enol ether cation radicals, in lieu of a detailed rate-order analysis.^32,34,35^ Having a reaction rate that is second order in methanol concentration implies that in addition to the methanol nucleophile acting as the trapping reagent, a second methanol molecule must serve as a catalyst, facilitating the reaction. After a methanol molecule traps the cation radical, its hydrogen atom becomes highly acidic, and the catalytic role of the second methanol likely involves this acidic proton. A corollary of this finding is that one might thus expect *intramolecular* alcohol trapping to be *first order* in methanol content (if methanol is solvent or cosolvent), if one assumes an analogous mechanism as for the methanol trapping process. This would seem commensurate with the large body of work by the Moeller group, which in numerous cases demonstrated particular yield sensitivity of anodic intramolecular alcohol coupling reactions to the solvent methanol content.^28,29^

In this work, we apply computational methods to investigate the catalytic role of methanol solvent in alcohol trapping of enol ether cation radicals, a critical step in certain intramolecular anodic coupling reactions. We investigate two different substrates, a 1,3,4,6-tetramethoxyhex-5-en-2-ol substrate (henceforth, tetramethoxyhexenol) and a 1-methoxy-1,6-octadiene (henceforth, methoxyoctadiene) substrate. Anodic intramolecular coupling of the first substrate results in tetramethoxyfuranose through a pathway involving oxidation of the enol ether group to a cation radical, followed by intramolecular nucleophilic attack by the hydroxyl group. It has been observed experimentally that the yield of this electrolysis depends sensitively on the electrochemical reaction conditions;^29^ for example, ∼85% yield was achieved with LiClO_4_/methanol electrolytes, with significantly reduced yield with either different salt (Et_4_NOTs) or solvent (THF) combinations. Our goal in this work is to explicitly characterize such solvent effects. Here, we compute reaction free energies for alcohol trapping of the cation radical intermediate, utilizing density functional theory (DFT)-based quantum mechanics/molecular mechanics (QM/MM) simulations. These simulations provide an atomistic description of the solvent, modulating the cation radical reaction described at a DFT level of theory. Reaction free energies are computed for both the target intramolecular alcohol trapping and competitive methanol trapping side reactions from the solvent. For the methoxyoctadiene substrate, a comprehensive computational study of the anodic intramolecular coupling reaction has been published recently by our group.^38^ Here, we compute and report reaction free energies for methanol trapping of the cation radical intermediate to complement this prior work.

Our computed DFT-based QM/MM free energy profiles unequivocally demonstrate the catalytic role of methanol in alcohol trapping of cation radical intermediates. Following nucleophilic attack by the alcohol, the acidic proton complexes with a methanol solvent molecule to form a “Zundel-like” intermediate, with the acidic proton shared between two oxygen atoms at 1.1–1.3 Å, O–H distances. This “shared proton” motif is crucial for forming the nucleophilic trapping intermediate, resulting in enhanced stabilization of ∼50–60 kJ/mol of the intermediate for both *intra*- and *inter*molecular alcohol trapping reactions. While the prior kinetic studies of Oyama et al. investigated different anthracene-derived cation radicals,^36,37^ the consistent conclusion of third-order kinetics for methanol or water trapping of cation radicals with our present study on enol ether cation radicals suggests a general mechanism by which cation radicals undergo nucleophilic attack by methanol/water solvent. Our results also suggest revised rate constants for prior work that assumed overall second-order kinetics for enol ether cation radical trapping reactions.^32,34,35^ Our computations strongly suggest that for the anodic, intramolecular coupling of tetramethoxyhexenol and similar substrates, the kinetics of the alcohol trapping step is first-order in methanol content of the electrolyte. This conclusion helps explain the observed electrosynthesis yield on reaction conditions^29^ and suggests that the electrochemical environment (e.g., electrical double layer) may play a crucial role in dictating competitive nucleophilic trapping reactions involving cation radicals and alcohols.

## 2 Methods

**Figure 1:**
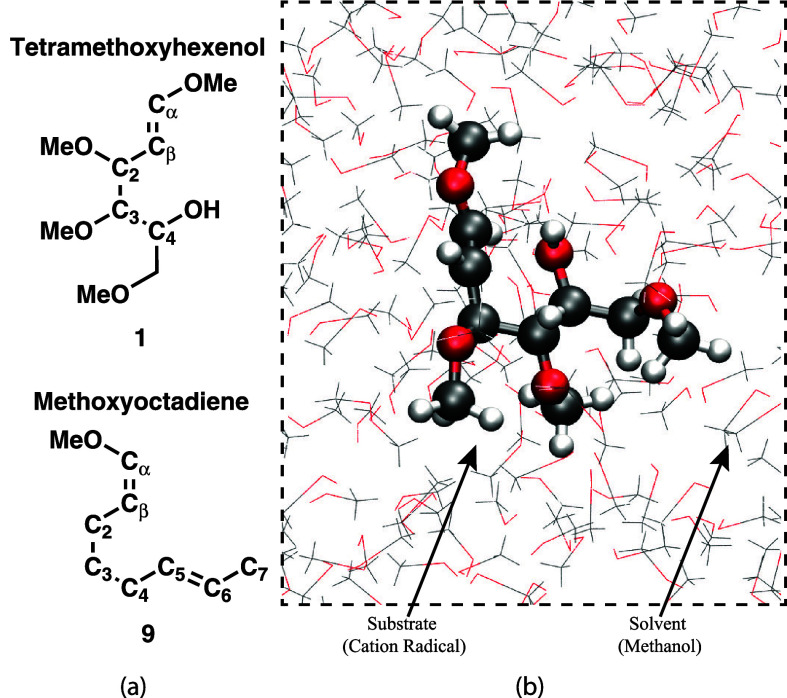
(a) Tetramethoxyhexenol and methoxyoctadiene substrates that are investigated as targets of anodic intramolecular coupling reactions; the atom labels used in the discussion of the free energy calculations are shown. The substrate labels (**1** and **9**) are consistent with labels from the respective reaction schematics in Figures 2 and 3 (b) Representative QM/MM simulation snapshot of the tetramethoxyhexenol substrate in bulk methanol.

DFT-based QM/MM free energy profiles for both intra- and intermolecular alcohol trapping reactions of the cation radical substrates were computed in bulk methanol solvent. The tetramethoxyhexenol (**1**) and methoxyoctadiene (**9**) substrates are depicted in Figure 1a, with a schematic of the solvated system in methanol shown in Figure 1b. The simulations consist of each substrate solvated in a box of 2000 methanol molecules and modeled with periodic boundary conditions. The initial configuration was set up using the PackMol software,^39^ followed by density equilibration of the system for 50 ns with classical molecular dynamics (MD) within the isothermal–isobaric (NPT) ensemble at 300 K and 1 bar. For the equilibration, the OPLS-AA force field^40^ was utilized for both the substrates and methanol solvent molecules. The force field parameters for the cation radical substrates are taken to be the same as their neutral counterparts (i.e., default OPLS-AA parameters), except for the charges. Atomic charges were parameterized for the cation radicals, based on DFT calculations at the PBE0/6-31G level of theory, followed by distributed multipole analysis (DMA)^41^ and subsequent atomistic charge fitting.^42,43^ Parameterized atomic charges for the +1 oxidation state of the substrates are given in Table S2. The classical MD simulations were performed with OpenMM v7.7 software,^44^ and utilized a time step of 1 fs, with Langevin thermostat and Monte Carlo barostat with frequencies of 1 ps^–1^ and 0.1 ps^–1^, respectively. Particle Mesh Ewald (PME)^45^ was utilized for electrostatic interactions, and a cutoff distance for van der Waals interactions was set at 1.4 nm. The equilibrated simulation box, with a dimension of 5.17 nm^3^, was used as the starting configuration for the subsequent QM/MM simulations within the NVT ensemble.

### 2.1 Quantum Mechanics/Molecular Mechanics Hamiltonian:

We employ a hybrid QM/MM framework^46−51^ to study the anodic coupling reaction mechanisms, specifically via QM/MM-MD simulations. The QM/MM methodology involves treating a region of chemical interest at a QM level of theory and the remaining chemically inert environment with a force field-based MM description. We model the QM region with a Kohn–Sham DFT Hamiltonian at the PBE0/6-31G level of theory with a 50/194 Lebedev-Treutler quadrature grid, and we model the MM region with the OPLS-AA force field.^40^ The QM region consisted of the cation radical substrate and additionally either zero, one, or two neighboring methanol molecules (as will be discussed below), with the remainder of the methanol solvent belonging to the MM region. QM/MM-MD simulations were performed using the PyDFT-QMMM simulation package,^52^ as interfaced with a modified version of the Psi4 quantum chemistry package (v1.5)^53^ and the OpenMM molecular dynamics package (v7.7).^44^ The QM and MM regions were coupled through a QM/MM/Cutoff scheme, consisting of electrostatic embedding of molecules in the MM region up to a group-based centroid cutoff of 1.4 nm evaluated at each time step and mechanical embedding of other nonbonded interactions via a Lennard-Jones (LJ) potential. The QM/MM-MD simulations were conducted in the NVT ensemble, utilizing a time step of 1 fs with a Langevin thermostat set at a temperature of 300 K, and a friction coefficient of 5 ps^–1^. At each time step, the self-consistent field (SCF) optimization of the Kohn–Sham QM/MM Hamiltonian utilized as an initial guess the converged wave function of the previous time step; unrestricted Kohn–Sham (UKS) was used to model the cation radicals’ doublet spin state. QM/MM-MD simulations were initialized from systems equilibrated from classical MD (see above); the QM substrate geometry was then replaced with optimized structure at the PBE0/6-31G level of theory, followed by subsequent equilibration of the full system with QM/MM-MD for 2 ps of simulation, preceding production simulations.

An important consideration is the applicability of the chosen level of DFT theory, PBE0/6-31G, for describing the cation radical reactions investigated in this work. There exists substantial literature documenting systematic errors in DFT predictions of the electronic structure (and, hence, energetics) of certain types of cation radicals.^54−58^ This results from the self-interaction error inherent to DFT methods,^58^ and can be partially mitigated with hybrid functionals that incorporate some amount of exact exchange.^57^ Specifically relevant to this work is the observation that DFT functionals tend to substantially overstabilize two-center, three-electron bonds of certain cation radical substrates.^56,57^ The cation radical substrates (oxidized **1** and oxidized **9**) investigated in this work likely fall within a similar category of systems for which the predictive accuracy of DFT is questionable. To address these concerns, we have conducted higher-level MP2/cc-pVTZ benchmarks for the intramolecular alcohol trapping of a cation radical substrate similar to the tetramethoxyhexenol cation radical (oxidized **1**), but chosen to be slightly smaller for computational tractability. These benchmarks are given in Figure S1, and the main takeaway is that the employed PBE0/6-31G DFT level of theory has expected errors of ∼5–10 kJ/mol in describing the energetics of these types of cation radical reactions. Changing to a larger basis set with polarization functions, e.g., PBE0/6-31G*, does not improve the predictive accuracy, given error cancellation (as discussed in the Supporting Information). We conclude that the PBE0/6-31G level of theory utilized within our QM/MM-MD simulations is thus sufficiently accurate for the purposes of this work because 1) the major investigation is solvent effects, for which relative energetic trends will be less affected by systematic error in the DFT functional, and 2) all qualitative conclusions are inferred from observed energetic differences that are significantly greater than the anticipated errors of ∼5–10 kJ/mol resulting from the DFT functional choice.

### 2.2 Free Energy Simulations:

QM/MM simulations with umbrella sampling are utilized to compute the cyclization and solvent attack reaction free energy surfaces. Three different reactions were investigated in bulk methanol: 1) intramolecular coupling of the tetramethoxyhexenol cation radical (oxidized **1**); 2) methanol trapping of the tetramethoxyhexenol cation radical (oxidized **1**); 3) methanol trapping of the methoxyoctadiene cation radical (oxidized **9**). Two-dimensional (2D) free energy surfaces were computed for all three reactions, utilizing umbrella sampling as performed with the PLUMED2 software.^59^ In all cases, the biasing umbrellas utilized force constants of 1200 kJ/mol/Å^2^, with umbrellas evenly spaced along the 2D coordinate grid at intervals of 0.1 Å. Each 2D free energy surface is generated from 400 umbrella simulations. Each umbrella simulation started from the same initial configuration, and a 2 ps steering trajectory was conducted, “pulling” the molecular configuration to the umbrella bias coordinate(s). Subsequently, a 25 ps trajectory (production) of DFT-QM/MM molecular dynamics was generated at each umbrella position, corresponding to 9.6 ns of total simulation time to construct each 2D free energy profile. The Weighted Histogram Analysis Method (WHAM) was used to reverse bias the distribution(s) to generate the free energy surface(s). The variables used to define the reaction free energy profiles are as follows: 1) *Intramolecular coupling of tetramethoxyhexenol cation radical* (oxidized **1**): Two distance coordinates are used, namely, C_α_-O_H_ and C_β_-O_H_, with labels of the carbon atoms depicted in Figure 1a. These two coordinates distinguish whether the intramolecular, nucleophilic attack occurs on cation radical C_α_ or C_β_ positions to form either six- or five-membered heterocycle products. 2) *Methanol trapping of tetramethoxyhexenol cation radical* (oxidized **1**): Two distance coordinates are used, namely, C_α_-O_Methanol_ and C_β_-O_Methanol_, to distinguish methanol solvent attack on the different radical cation configurations. For each of these distance variables, the 2D reaction coordinate spans a range of 1.3 Å to 3.2 Å with a spacing of 0.1 Å. 3) *Methanol trapping of methoxyoctadiene cation radical* (oxidized **9**): Two distance coordinates are used, namely, C_β_-O_Methanol_ and C_β_-C_6_, with labels of the carbon atoms depicted in Figure 1a. These two coordinates elucidate how solvent attack is coupled to the olefin cyclization reaction. The C_β_-C_6_ coordinate spans 1.5 Å to 6.0 Å with a spacing of 0.1 Å, while the C_β_-O_Methanol_ coordinate spans 1.4 Å to 3.35 Å with a spacing of 0.1 Å.

Reaction free energies were computed with different selections of methanol solvent molecules to include in the “QM” region. In all cases, the radical cation substrate (either oxidized **1** or oxidized **9**) is included in the “QM” region, and zero, one, or two additional methanol solvent molecules are also included in the QM region. The specific choice of QM region is a central aspect of the study, and will be explicitly discussed in the context of the presented results. When methanol solvent molecules are included in the QM region, the molecules chosen are those in close proximity to the radical cation substrate that may participate in the chemical reaction. Solvent molecules outside the QM region, as described at an “MM” level of theory, modulate the electronic structure of the QM/reactive region through electrostatic embedding of their atomic charges but cannot participate in chemical bonding or orbital interactions. We will elaborate on these differences while discussing the results.

## 3 Results and Discussion

Our study focuses on the catalytic role of methanol solvent in alcohol trapping of cation radical intermediates, oxidized **1** and oxidized **9**. This is a key step in certain anodic coupling reactions, but there are several other electrochemical steps in the full electrosynthesis process that are not discussed in detail in this work. In Section 3.1, we give an overview of the full electrosynthesis process for the target substrates investigated in this work to provide context for the computational characterization of alcohol trapping reactions. This discussion is based on intramolecular, anodic coupling reactions previously performed by Moeller and coworkers on the tetramethoxyhexenol (**1**) and methoxyoctadiene (**9**) substrates.^13,26,29^ In Sections 3.2–3.4, we then discuss our computational results for intra- and intermolecular alcohol trapping reactions with the cation radical substrates/intermediates.

### 3.1 Electrosynthesis: Anodic, Intramolecular Coupling Reaction Mechanisms

**Figure 2:**
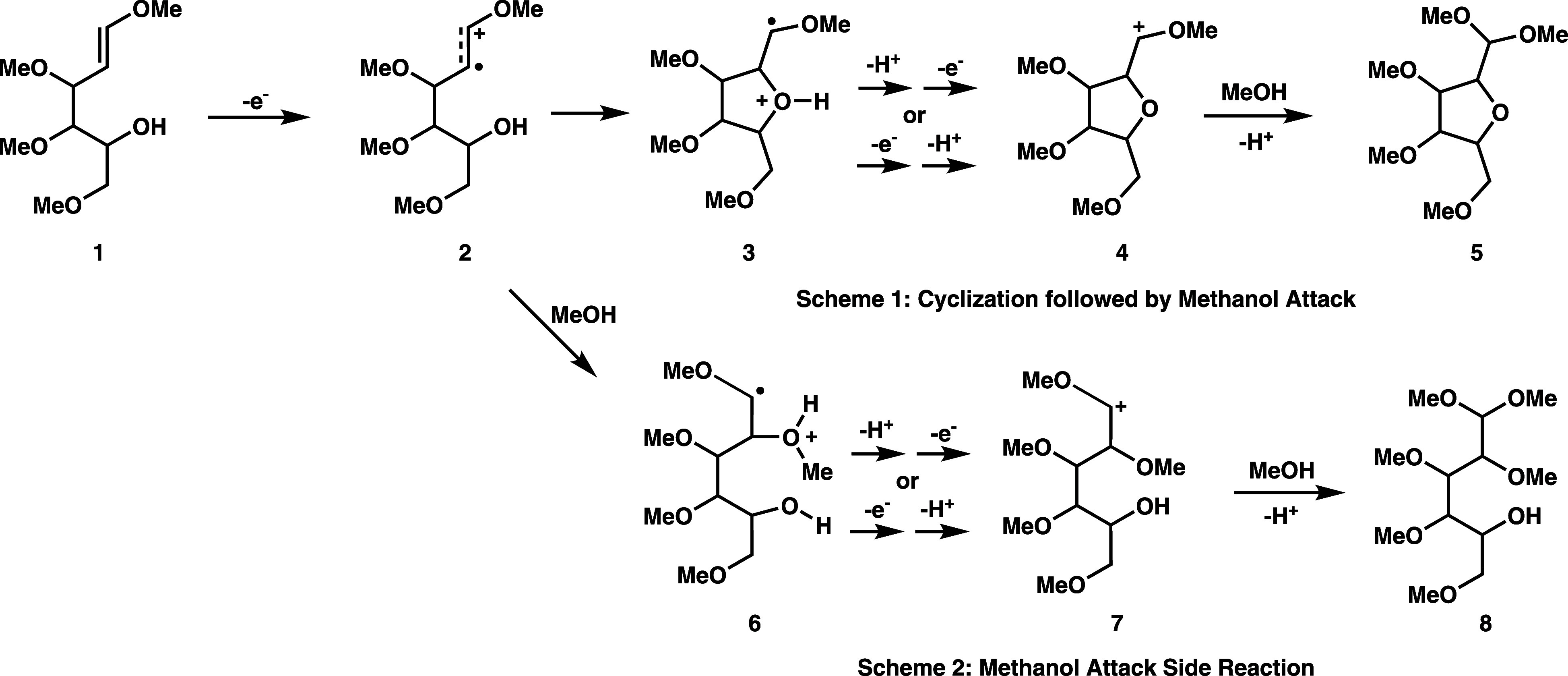
Reaction diagrams for anodic, intramolecular coupling of tetramethoxyhexenol substrate (**1**) showing target heterocycle formation (**5**) in Scheme 1, and a possible methanol attack side reaction of the cation radical intermediate in Scheme 2.

We focus on two substrates that were the target of anodic, intramolecular coupling electrosynthesis reactions previously conducted by Moeller and coworkers.^13,26,29^ The first reaction is shown in Figure 2, which involves the electrosynthesis of a tetramethoxyfuranose derivative (**5**), starting from the tetramethoxyhexenol (**1**) substrate.^29^ Upon oxidation, the key intramolecular coupling reaction involves the enol ether initiator and the hydroxyl group on the opposite end of the substrate. Anodic oxidation at the enol ether functional group produces the cation radical intermediate **2**. Intramolecular nucleophilic attack by the hydroxyl group forms the heterocyclic cation radical **3** (Scheme 1, Figure 2); presumably, cyclization of the cation radical intermediate **3** is reversible.^60^ Loss of a proton and a second oxidation produces a heterocyclic cation intermediate **4**, and subsequent methanol trapping (and another deprotonation) gives the final furanose derivative product **5** (Scheme 1). Overall, the final product formation involves two oxidations, two nucleophilic trappings, and two deprotonation steps.^61,62^ In Figure 2, we indicate two possibilities for the first deprotonation step, occurring either before or after the second oxidation; this is based on the computed second oxidation potential of the cyclized cation radical structure **3** (i.e., no deprotonation), being of similar magnitude to the first oxidation potential of the substrate (Supporting Information).

A potential side reaction pathway is shown in Scheme 2 of Figure 2. Following oxidation of enol ether **1**, cation radical **2** could instead be trapped by a methanol solvent molecule. Deprotonation, followed by a second oxidation, would then lead to an acyclic cation intermediate, and trapping by a second methanol molecule (and subsequent deprotonation) would lead to the acyclic side product **8** shown in Scheme 2. Clearly, the propensity for a plausible side reaction pathway involving methanol trapping, relative to the intended heterocycle formation of **5** via intramolecular alcohol trapping, would be dependent on the methanol content of the electrolyte. In the electrosynthesis experiments of Moeller and coworkers,^29^ different electrolytes were tested, consisting of either pure MeOH solvent or MeOH/THF cosolvent, with either LiClO_4_ or Et_4_NOTs salts. The observed yield of the target tetramethoxyfuranose product (**5**) was highly dependent on the employed electrolyte, with the highest ∼85% yield achieved with LiClO_4_/MeOH electrolyte. Moeller and coworkers attributed the substantial variation in yield to an influence of the electrical double layer, whereby a “greasier” Et_4_NOTs double layer might prevent the approach of the tetramethoxyhexenol (**1**) substrate to the electrode surface.^29^ In Sections 3.3 and 3.4, we present reaction free energy computations for the separate alcohol trapping steps of Schemes 1 and 2 (Figure 2) to provide further insight concerning the role of methanol in the electrosynthesis process.

**Figure 3:**
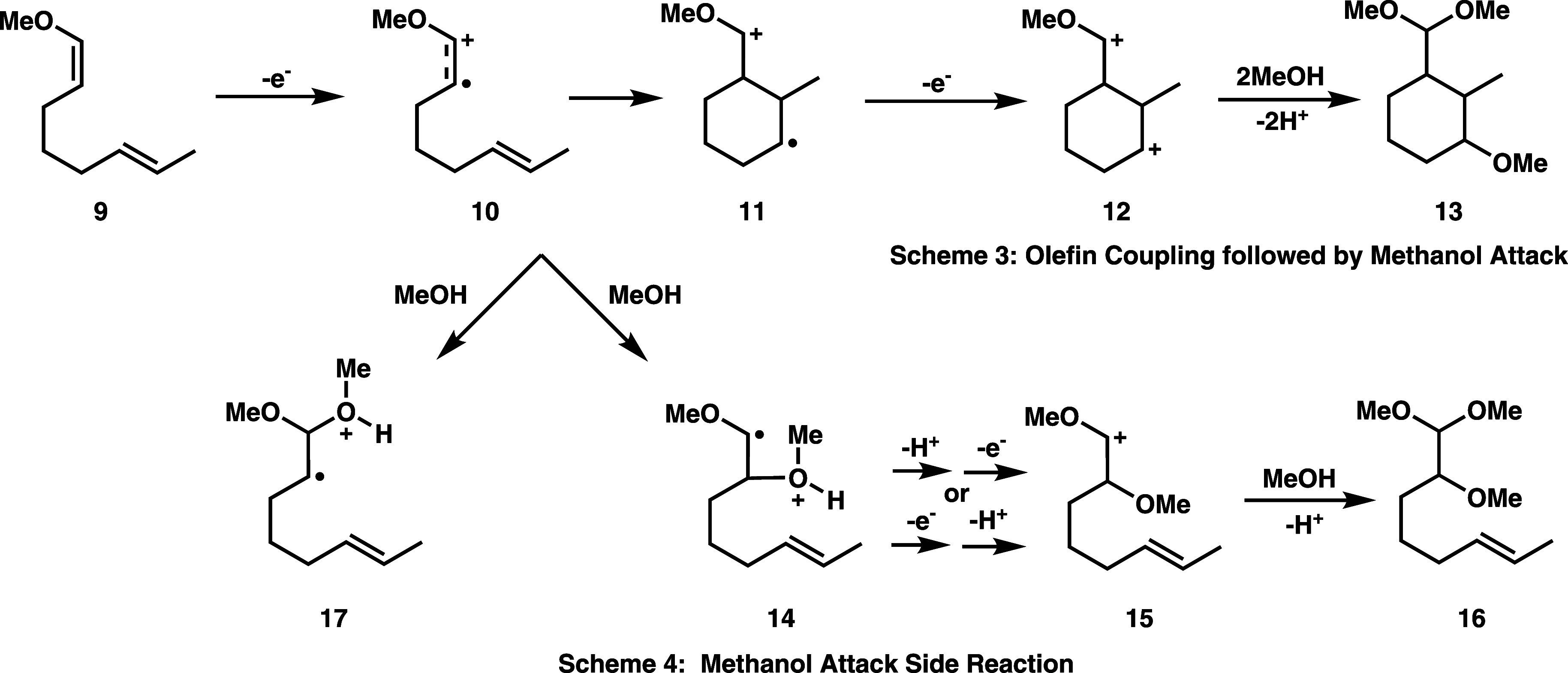
Reaction diagrams for anodic, intramolecular coupling of methoxyoctadiene (**9**) substrate showing target cyclized product **13** formation in Scheme 3 and possible methanol attack side reaction of the cation radical intermediate in Scheme 4.

The second electrosynthesis reaction that we consider is shown in Figure 3. This involves anodic oxidation of a methoxyoctadiene substrate **9**, followed by intramolecular cyclization to either a 5-member or 6-member ring; only the 6-member ring product **13** is shown in Figure 3, but 5-member ring products are also experimentally observed.^13,26^ This intramolecular coupling reaction, shown in Scheme 3 of Figure 3, is similar to that discussed in Scheme 1 of Figure 2, except that a second olefin of **10,** rather than a hydroxyl group, serves as the nucleophile for the cyclization step. Cyclization of the cation radical **10** in Scheme 3 is reversible, and only after a second oxidation to the dication **12** is the cyclization irreversible, and the regiochemistry (e.g., 5-member vs 6-member ring) locked in.^13^ The final (neutral) cyclization product **13** is then formed by methanol trapping of the dication and subsequent deprotonation, as shown in Scheme 3. In a prior computational study, we have computed free energies of several key steps for the electrosynthesis reaction shown in Scheme 3, within the electrochemical environment.^38^ Our study suggested that electrostatic stabilization of the cyclized cation radical intermediate **11** within the electrical double layer may significantly contribute to the observed product yield.^38^

Our prior work did not investigate possible methanol trapping side reactions. In Scheme 4 of Figure 3, we show such a plausible side reaction, which is similar to the methanol trapping reaction shown in Scheme 2 of Figure 2. In Scheme 4, methanol trapping of the cation radical intermediate **10**, followed by deprotonation, a second oxidation, and trapping by a second methanol molecule, forms a side product **16** that precludes the intended intramolecular cyclization. The electrosynthesis shown in Figure 3 has been experimentally conducted with LiClO_4_/MeOH electrolyte (among other choices)^13,26^ so that the methanol trapping side reaction depicted in Scheme 4 would seem plausible. Because of the demonstrated efficacy for alcohols to trap enol ether cation radicals, one may question whether the target anodic cyclization reaction shown in Scheme 3 would achieve higher yield in a low-methanol content electrolyte to minimize methanol trapping side reaction(s).^29^ In Section 3.4, we present reaction free energy computations for the methanol trapping side reaction of Scheme 4 (Figure 3), which complements our prior free energy computations^38^ for the electrosynthesis reaction shown in Scheme 3.

**Figure 4:**
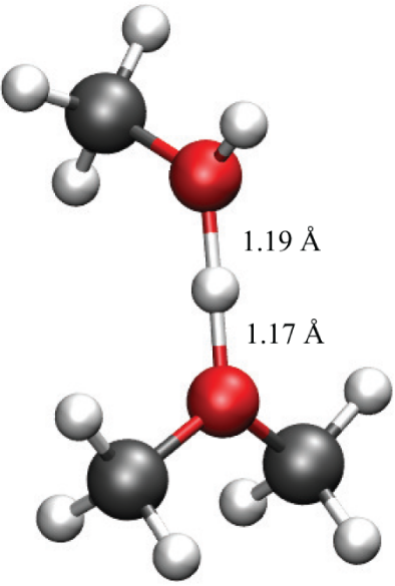
Simplified model of the oxonium cation complex formation with methanol. Calculations are done at the PBE0-D3/6-31G level of theory with the conductor-like polarizable continuum model (CPCM) for methanol.

Before our computational results are presented, it is worthwhile to consider a hypothesis for how/why methanol may have a catalytic role in the alcohol trapping reactions discussed above. Alcohol trapping of an enol ether cation radical will result in a highly acidic proton, and it is expected that the positive charge of the substrate will be localized on this −OH group, as shown schematically in Figures 2 and 3. This positively charged −OH group will have substantial interaction with a hydrogen-bond acceptor, such as the oxygen atom of a methanol solvent molecule. As an analogy, this situation is quite similar to the solvation of hydronium ions in water; a hydronium ion exhibits the largest solvation energy of any common monovalent cation,^63^ due in large part to the very strong interaction of its protons with oxygen atoms of solvating water molecules.^64^Figure 4 provides a concrete illustration of such a motif, depicting a model oxonium cation complexed with a methanol solvent molecule. From DFT calculations in an implicit (methanol) solvent, the oxonium cation/methanol complex is energetically stabilized by ∼23 kcal/mol relative to the separated species. In this complex, the proton is shared between the two oxygen atoms, with both O–H bond distances being ∼1.2 Å. The geometry and O–H distances are very similar to those of the “Zundel”  motif of a hydronium ion solvated in water.^64,65^ The motif in Figure 4 suggests the substantial importance of solvent stabilization of the radical cation intermediates (with acidic protons), as shown in Figures 2 and 3.

The above hypothesis of solvent complexation/stabilization for alcohol trapping of cation radical intermediates would manifest in specific rate order dependence on methanol concentration. Two different rate order equations may be proposed for the cases of *intra-* and *inter*molecular alcohol trapping steps shown in the electrosynthesis reaction mechanisms of Figures 2 and 3. For *intra-* molecular, alcohol trapping such as in Scheme 1 of Figure 2, it is proposed that stabilization of the heterocyclic cation radical intermediate **3** (i.e., formed after alcohol attack) requires complexation with a methanol solvent molecule to form a motif similar to Figure 4. This would give overall second-order kinetics, with a rate that is first order in the cation radical substrate concentration and additionally first order in methanol concentration, i.e.
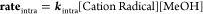
1with *k*_intra_ being the rate constant for the intramolecular alcohol trapping of the specific cation radical substrate.

For *inter*molecular alcohol trapping, such as in Scheme 2 of Figure 2 or Scheme 4 of Figure 3, one solvent methanol molecule serves as the nucleophile to trap the cation radical substrate (e.g., **2** or **10**). Consistent with the above hypothesis, a second methanol molecule would be required to complex with the positively charged −OH group to form a structural motif similar to Figure 4. This would give overall third-order kinetics, with a rate that is first order in the cation radical substrate concentration and second order in methanol concentration, i.e.
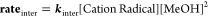
2with *k*_inter_ being the rate constant for the intermolecular methanol trapping of the specific cation radical substrate.

As noted in the introduction, kinetic studies by Oyama et al. have experimentally determined rate orders consistent with eq 2 for methanol or water trapping of anthracene-based cation radicals.^36,37^ As we will discuss in Section 3.4, our free energy computations for the alcohol trapping steps of reactions in Figures 2 and 3 unequivocally support the kinetic rate orders given by eqs 1 and 2 for intra- and intermolecular alcohol coupling, respectively, of enol ether radical cations. In other words, the key radical cation intermediate is a solvent-complexed structure analogous to the motif shown in Figure 4.

### 3.2 Geometric Conformations of the Cation Radical Substrates

**Figure 5:**
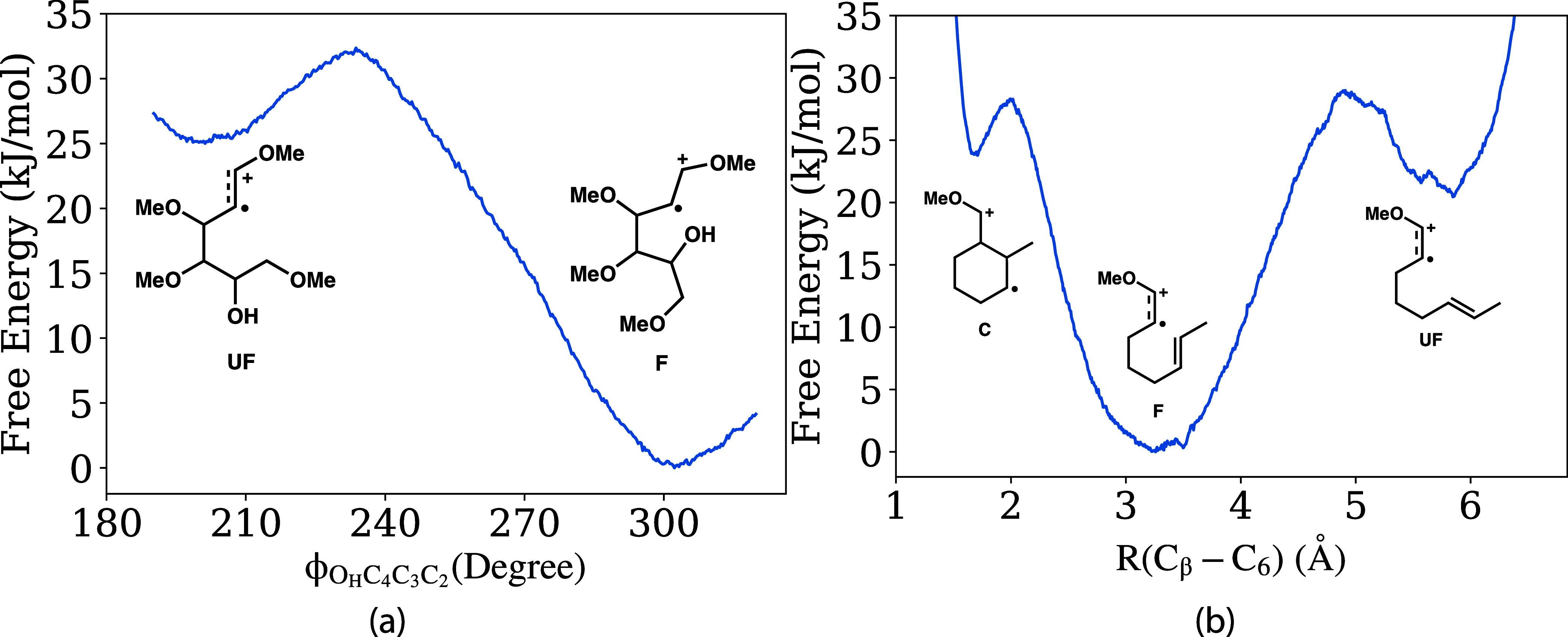
(a) Gas-phase free energy profile of the oxidized tetramethoxyhexenol substrate **2** computed along the dihedral rotation of the O_H_–C_4_–C_3_-C_2_ backbone (b) Gas phase free energy profile of the oxidized methoxyoctadiene substrate **10** computed along the C_β_-C_6_ distance coordinate. See Figure 1a for a definition of the atom labels.

The cation radical intermediates **2** and **10** of the electrosynthesis reactions shown in Figures 2 and 3 have an energetic preference for specific geometric conformations, as relevant to nucleophilic trapping. To illustrate this, we compute conformational free energies as a function of specific coordinates for the tetramethoxyhexenol (**2**) and methoxyoctadiene (**10**) cation radical intermediates. These free energies are computed in the gas phase (i.e., no methanol solvent) with DFT at the PBE0/6-31G level of theory, utilizing molecular dynamics simulations in combination with umbrella sampling, with details similar to as described in Section 2. Figure 5a,b depict the conformational free energy profiles for the tetramethoxyhexenol (**2**) and methoxyoctadiene (**10**) radical intermediates, respectively. We note that the methoxyoctadiene cation radical free energy profile in Figure 5b is identical to that reported in our prior work,^38^ and is reproduced here for completeness. For the tetramethoxyhexenol substrate **2**, the free energy profile is computed as a function of the coordinate of the O_H_–C_4_–C_3_–C_2_ dihedral (Figure 5a). For the methoxyoctadiene substrate **10**, the free energy profile is computed as a function of the distance between the C_β_ and C_6_ carbon atoms (Figure 5b). These specific coordinates are chosen to distinguish conformations for when the alcohol/olefin nucleophile is in close or far proximity to the oxidized enol ether cation radical.

Both cation radical substrates exhibit substantial free energy stabilization when the nucleophile (either alcohol or olefin group) is positioned in close proximity to the oxidized enol ether group. In prior work we termed such configurations “folded”,^38^ since the geometries represent a precyclized state (but without bond formation), and we use the same terminology here as labeled “F” in the free energy profiles of Figure 5. When the nucleophile (either alcohol or olefin group) is far from the oxidized enol ether group (according to the coordinates represented in Figure 5), the conformations are termed “unfolded”, with geometries that are not in direct position for intramolecular nucleophilic attack, as labeled “UF” in the free energy profiles of Figure 5. There exist “UF” and “F” local minima on the free energy profile of both cation radical substrates, but in both cases the folded conformation (F) is the global free energy minimum, being ∼20–25 kJ/mol more stable than the unfolded (UF) conformation depending on the substrate. The stability of the folded configuration for the methoxyoctadiene cation radical substrate **10** was previously rationalized by π–π orbital interactions between the olefin nucleophile and the oxidized enol ether electrophile.^38^ Similarly, the stability of the folded configuration for the tetramethoxyhexenol cation radical substrate **2** is due to orbital overlap between the lone pair of the O_H_ and the oxidized enol ether group, as shown in the Figure S2. It is important to note that while the folded conformations have precyclized geometries, they are not cyclized structures in that chemical bonds have not been formed between nucleophilic and electrophilic groups; e.g., there is ∼3.3 Å separation between the relevant carbon atoms and 2.2 Å separation between the oxygen and carbon atom involved in bond formation for the respective cyclization products (Figure 5).

The free energy profiles in Figure 5 indicate that cyclized conformations are unstable in the gas phase for both tetramethoxyhexenol (**2**) and methoxyoctadiene (**10**) cation radical substrates. For the methoxyoctadiene cation radical **10**, the cyclized conformation (denoted “C” in Figure 5b) is indeed a local minimum in the gas-phase free energy profile, but it is higher in energy by ∼23 kJ/mol compared to the folded “F” global minimum conformation. However, as discussed in previous work,^38^ the charge (de)localization of the radical cation **10** changes with conformation, so that electrostatic interactions within the electrochemical environment may play an important role in stabilizing the cyclized cation radical intermediate **11**, and thus influencing the overall yield of the electrosynthesis reaction (Figure 3). For the free energy profile of the tetramethoxyhexenol cation radical **2**, the O_H_-C_4_–C_3_-C_2_ dihedral coordinate does not distinguish between folded “F” and corresponding cyclized “C” conformations (the latter with a fully formed bond). However, we have performed accompanying gas-phase electronic structure calculations to verify that the cyclized conformation is not stable in the gas phase (i.e., there is no “C” local minimum). This is consistent with prior DFT calculations of Campbell et al. that reported no stable heterocyclic conformation for a similar cation radical substrate.^61^ The observation of no stable heterocycle formation for the tetramethoxyhexenol cation radical **2** in the gas phase, is consistent with the posed hypothesis in Section 3.1 that solvent/methanol complexation is required to stabilize the heterocyclic radical cation intermediate **3**.

### 3.3 Intramolecular Alcohol Coupling of Cation Radical Substrates

The discussion in Section 3.2 strongly suggests that the solvation environment plays a crucial role in the electrosynthesis reactions of Schemes 1 and 3 (Figures 2 and 3). The cyclized cation radicals **3** and **11** are key intermediates in the reaction mechanisms but have limited stability in the gas phase, and thus their formation likely requires solvent stabilization. The solvation environment within an electrosynthesis reaction is complex, as the substrate transitions from the electrical double layer near the electrode surface (i.e., for initial oxidation) back to the bulk electrolyte during the course of the full electrochemical process. Empirical evidence suggests that the anodic electrical double layer environment has an important influence on the product yield of the electrosynthesis reaction shown in Scheme 1 (Figure 2).^29^ While we have previously studied the role of the double layer on the reaction in Scheme 3,^38^ here for simplicity, we do not consider the complexity of the electrical double layer environment. Rather, since the focus is on the catalytic role of methanol, we investigate the reactions within bulk methanol solvent, as described in Section 2. As we will show, this model solvation environment of bulk methanol is sufficient for demonstrating the primary role of methanol solvent in catalyzing alcohol coupling reactions of cation radical substrates **2** and **10**.

**Figure 6:**
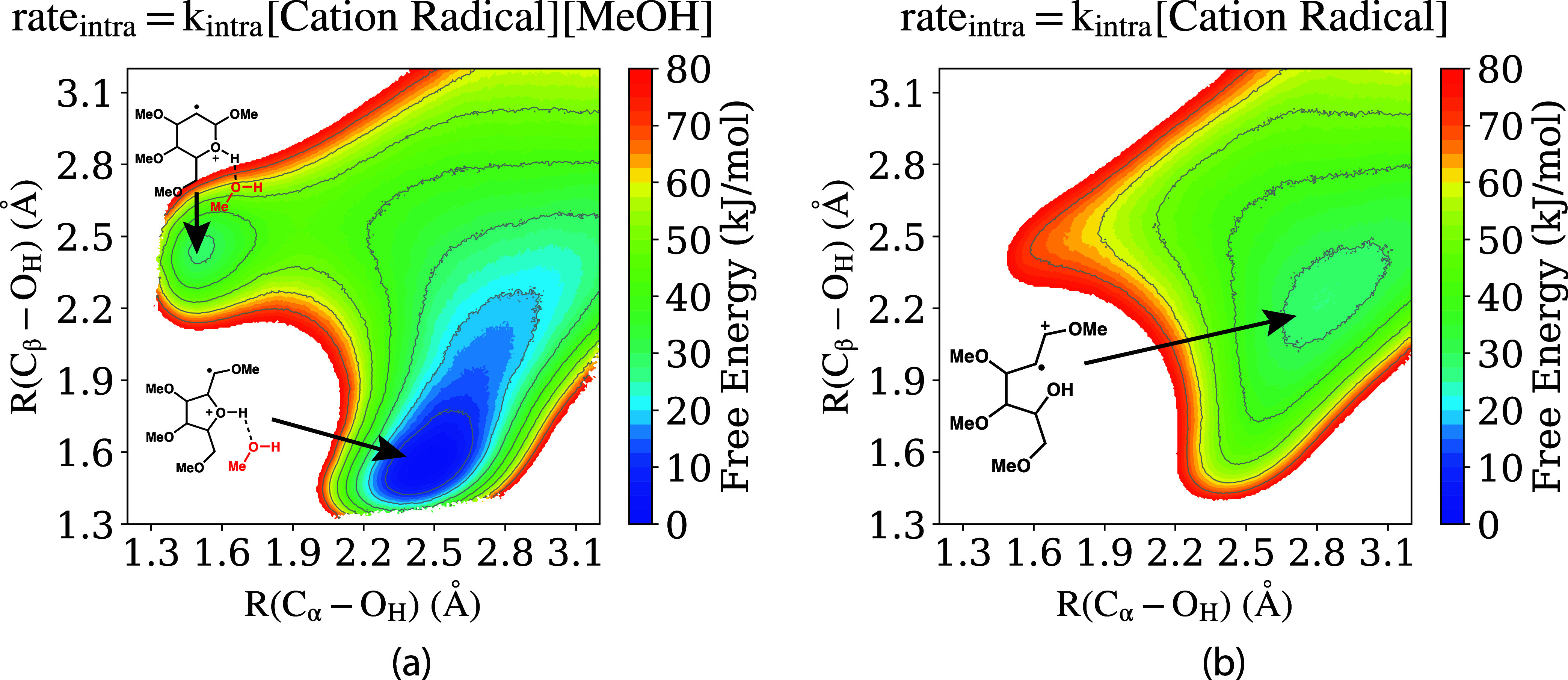
Free energy surfaces for intramolecular cyclization of the tetramethoxyhexenol radical cation substrate **2**, within bulk MeOH solvent. The contour plots are depicted with a change of color every 2.5 kJ/mol interval and bold contour lines every 10 kJ/mol interval. (a) Computed from QM/MM simulations with a close-contact methanol molecule in addition to the radical cation substrate included in the “QM” region; the cyclized cation radical structure **3** corresponds to the deep (blue) minimum. (b) Computed from QM/MM simulations with only the radical cation substrate included in the “QM” region.

We first discuss the intramolecular alcohol coupling reaction of the tetramethoxyhexenol (**2**) radical cation, leading to the cyclization intermediate **3** shown in Scheme 1 (Figure 2). As described in Section 2, the free energy of this reaction was computed with DFT-based QM/MM molecular dynamics simulations in bulk methanol. The 2D reaction free energy surface is described by the two distance coordinates, R(C_α_–O_H_) and R(C_β_–O_H_), between the oxygen atom of the −OH nucleophile and the two electrophilic carbon atoms of the oxidized enol ether group. As discussed in Section 2, separate QM/MM simulations were conducted with/without methanol solvent molecule(s) in the “QM” region in addition to the cation radical substrate (**2**). We first discuss the case in which one methanol molecule along with the cation radical substrate **2** is chosen as the “QM” region. The reaction free energy computed from this simulation is shown in Figure 6a.

Figure 6a presents the free energy surface for the intramolecular cyclization reaction with a quantum mechanical description of the radical cation substrate **2** and one (close-contact) methanol molecule, within bulk methanol solvent. The plot ranges from 1.3 to 3.2 Å, spanning both R(C_α_ – O_H_) and R(C_β_ – O_H_) distances on the *x*-axis and *y*-axis, respectively. There are two local minima on the free energy surface: the first is centered at R(C_α_–O_H_) ∼ 1.5 Å and R(C_β_–O_H_) ∼ 2.4 Å, and corresponds to the formation of a 6-membered ring, as depicted in the figure. The second minima is centered at R(C_β_–O_H_) ∼ 1.5 Å and R(C_α_– O_H_) ∼ 2.5 Å, and corresponds to the formation of a 5-membered ring, as also depicted in the figure. For comparison, the folded “F” conformer that is the global (free) energy minimum for the cation radical substrate in the gas phase (Figure 5) is located at approximately R(C_α_–O_H_) ∼ 3.0 Å and R(C_β_–O_H_) ∼ 2.3 Å on the 2D free energy surface in Figure 6a.

In light of the fact that no stable cyclized structure is observed in the previously discussed gas-phase quantum chemistry calculations (Figures 5), Figure 6a indicates that methanol solvent provides crucial stabilization of the cyclized tetramethoxyhexenol (**3**) radical cation conformer, which is a key intermediate in the overall electrosynthesis reaction mechanism (Scheme 1 of Figure 2). Within methanol solvent, the 5-membered ring conformer is the global free energy minimum for the radical cation substrate, being ∼20 kJ/mol more stable than the folded “F” conformer. Furthermore, the cyclization is a barrierless process within methanol (at least from the folded intermediate), with an entirely downhill free energy path between the “F” and 5-membered ring conformers. It is interesting that a 6-membered ring structure is also observed as a local free energy minimum in Figure 6a, given that a corresponding 6-membered heterocyclic product was not observed in the electrosynthesis experiments.^29^ Despite being a local free energy minimum conformation, the lack of experimentally observed 6-membered ring product is rationalized based on kinetics. There is a significant ∼25 kJ/mol barrier separating the 6-membered ring and folded “F” conformer. In contrast, the conformational transition to the 5-membered ring from the “F” conformer is barrierless, and additionally, the 5-membered radical cation cyclized intermediate **3** is more stable by ∼25 kJ/mol compared to the 6-membered ring.

The stability of the 5-membered ring conformer for the tetramethoxyhexenol radical cation intermediate (Figure 6a) relies on complexation of a methanol solvent molecule with the oxonium ion motif of the cyclized structure, analogous to Figure 4. This complexation is a partially covalent orbital interaction, with the corresponding O–H distance much shorter than for a typical hydrogen bond and/or noncovalent interaction. The interaction thus cannot be captured by a standard molecular mechanics force field or “MM” interaction within the QM/MM framework and must be described at the DFT (or other quantum mechanical) level. The QM/MM simulations generating the free energy profile in Figure 6a modeled the close-contact methanol and radical cation substrate at the DFT (“QM”) level, such that this cation/methanol complexation was captured. For comparison, in Figure 6b, we present the analogous free energy profile, but with only the cation radical substrate **2** included in the “QM” region (DFT) and all methanol solvent molecules modeled at the “MM” level. As evident, the free energy profiles in Figure 6a,b are distinctly different; the profile in Figure 6b shows no stable 5-membered ring conformer **3**, due to the fact that methanol complexation does not occur when the interaction is not described at a quantum mechanical level. We note that the energy scale has been adjusted for Figure 6b in order to compare with Figure 6a. For Figure 6a, considering the fact that at the geometric configuration R(C_α_–O_H_) ∼3.2 Å and R(C_β_–O_H_) ∼3.2 Å, methanol is not coordinating (i.e., no complex formation) with the −OH group. So, the relative energies of the top-right points have been set equal for Figure 6a,b.

**Figure 7:**
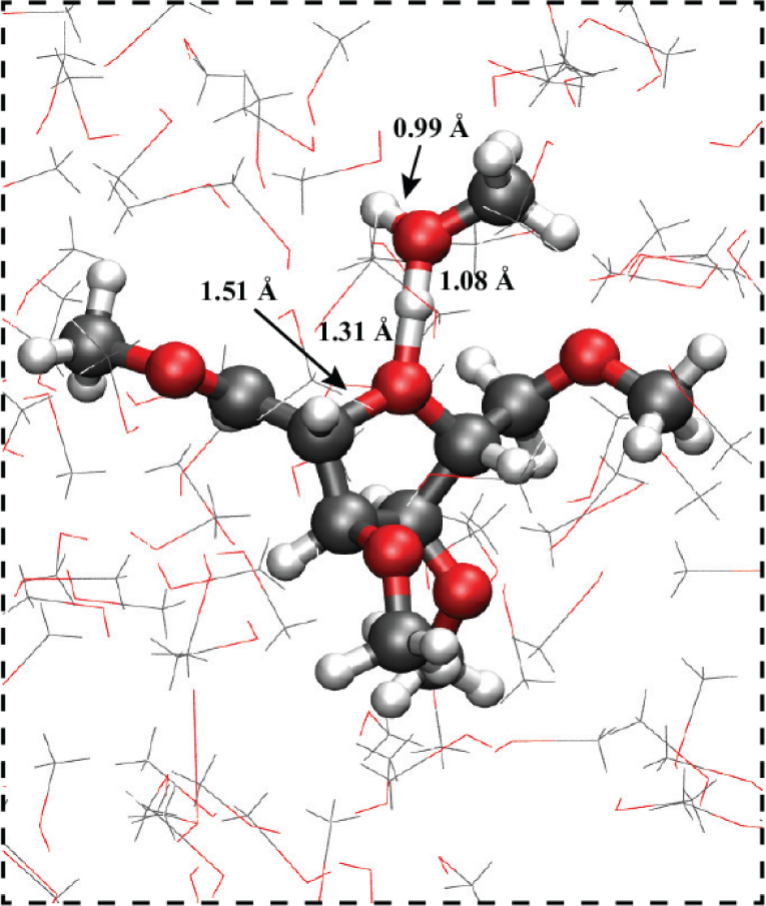
QM/MM simulation snapshot of the cyclized tetramethoxyhexenol radical cation **3** substrate in bulk MeOH solvent. The snapshot is from the simulation consistent with the free energy profile in Figure 6a, in which the QM region is composed of the closest methanol molecule in addition to the cation radical substrate **3**.

Figure 7 shows a snapshot from the QM/MM simulations that elucidates the methanol/cation radical complexation in more detail. The snapshot is from the simulation consistent with the free energy profile in Figure 6a, in which the QM region is composed of the closest methanol molecule in addition to the cyclized cation radical substrate **3**. The snapshot depicts the 5-membered, cyclic conformer, corresponding to the global free energy minimum in Figure 6a. Complexation of the cyclized cation radical substrate **3** with a close methanol solvent molecule is clearly observed. For this structure, the newly formed C_β_-O bond is 1.51 Å, with O–H bond distances of 1.31 and 1.08 Å between the shared proton and oxygen atoms of the cation radical substrate **3** and methanol. This complexed structure is similar to the oxonium cation/methanol motif shown in Figure 4, except that here, the O–H distances are asymmetric, with a shorter bond distance between the proton and the methanol oxygen atom. In Figure S3, we plot the distribution of the two O–H bonds from the QM/MM simulation, as computed for cyclic radical cation structure **3** complexed with methanol. The snapshot in Figure 7 is representative of the center of the distribution; on average, the two O–H bonds are ∼1.1 and ∼1.3 Å, with the shorter distance for the methanol oxygen atom. The bond distance fluctuations are ∼0.1–0.2 and ∼0.2–0.3 Å, with smaller fluctuations for the O–H bond involving the methanol oxygen atom (Figure S3). During the course of the QM/MM simulation, the cation radical substrate does not deprotonate; rather, it remains complexed with this methanol solvent molecule. This indicates that the complex is a (meta-)stable intermediate, at least on the time scales of the simulation.

The reaction free energies presented in Figure 6 strongly support the postulate of second-order kinetics (eq 1) for the intramolecular alcohol coupling reaction of the cation radical substrate **2**. As indicated by the title captions on the graphs, Figure 6a,b essentially correspond to cyclization reactions proceeding through overall second- or first-order kinetics, respectively. Without a methanol molecule participating in the complexation of the radical cation to stabilize the acidic proton, the cyclic structure is unstable by ∼30–35 kJ/mol relative to the folded “F” intermediate (Figure 6b). If the cyclic cation radical structure **3** is unable to form, deprotonation of the −OH group and/or a second oxidation cannot occur (given no overpotential and neutral pH conditions), and the electrosynthesis reaction mechanism (Scheme 1, Figure 2) cannot proceed. In contrast, when the reaction occurs via second-order kinetics (i.e., first order in methanol), the key cyclized intermediate **3** is readily formed/stable due to the methanol complexation (Figure 6a).

We finally comment on the subtle interpretation of the QM/MM simulation results of Figure 6b. While this QM/MM simulation was performed in bulk methanol, it provides an unphysical description of the cyclization reaction within methanol solvent, since the MM modeling of methanol molecules does not capture the important methanol/cation radical complexation. Thus, the free energy profile of Figure 6b is more representative of the reaction within a different solvent environment, for instance, possibly acetonitrile, with molecules that do not complex with the acidic proton of the cyclic cation radical substrate **3**. Note that electrostatic interactions between the cation radical substrate **3** and methanol solvent *are* indeed fully captured in the QM/MM simulation corresponding to Figure 6b, but the requisite complexation motif (Figure 7) represents a quantum mechanical (i.e., covalent) interaction and is not simply classical electrostatics.

### 3.4 Methanol Attack to the Cation Radical Substrates

The results of Figure 6a demonstrate that intramolecular alcohol trapping of the oxidized enol ether group is a highly favorable and barrierless reaction via complexation with a methanol solvent molecule. This would imply that the oxidized enol ether group is susceptible to alcohol attack by the methanol solvent itself. Correspondingly, as indicated in Figures 2 and 3, methanol solvent attack is expected to be a competitive side reaction with the desired anodic, intramolecular coupling pathways of both the tetramethoxyhexenol (**2**) and methoxyoctadiene (**10**) substrates. Analogous to Section 3.3, here we perform DFT-based QM/MM simulations to compute reaction free energies for methanol trapping of the cation radical substrates **2** and **10**.

**Figure 8:**
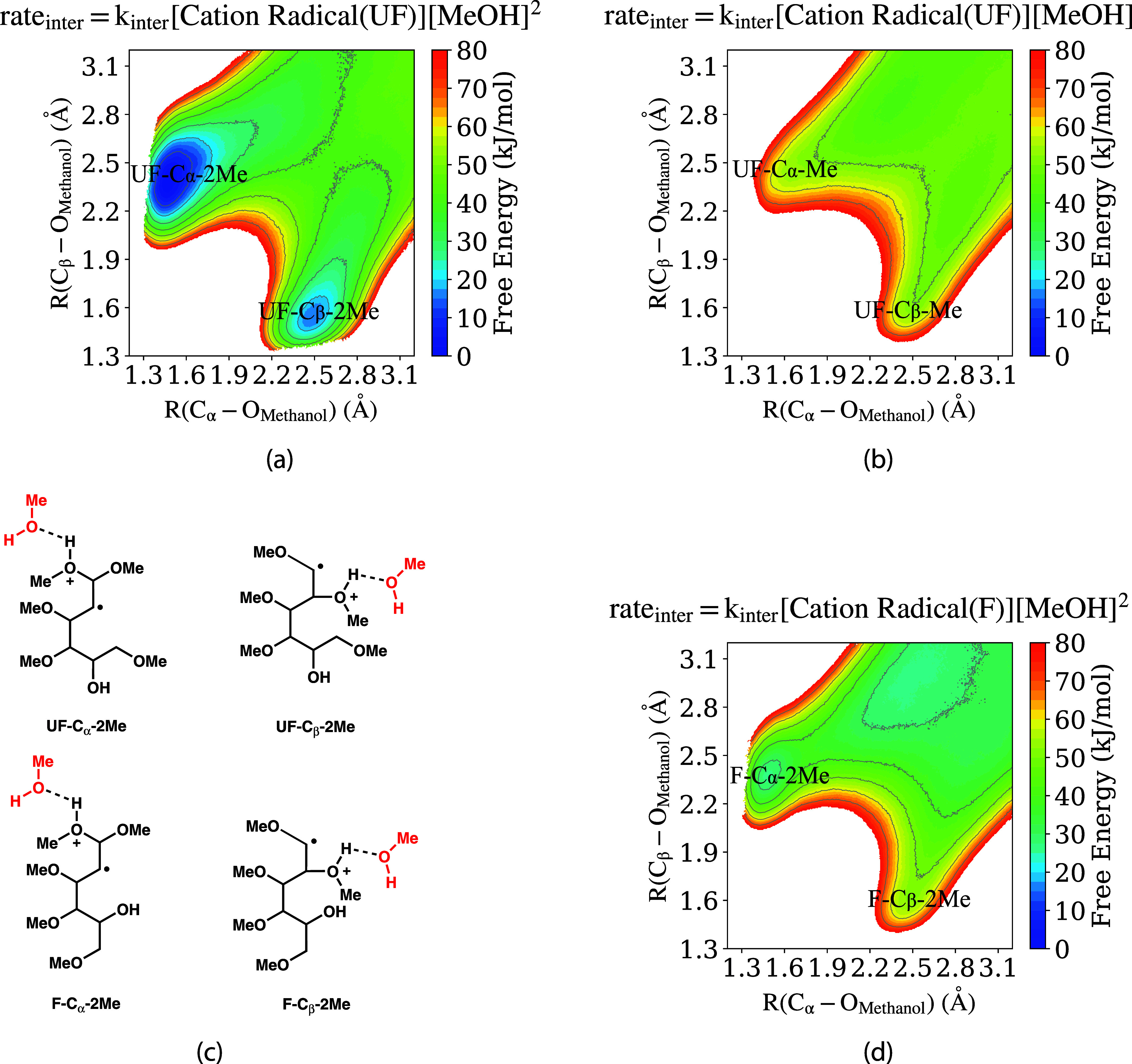
2D free energy surfaces for methanol attack to tetramethoxyhexenol cation radical substrate **2** at both C_α_ and C_β_ sites (see Figure 1 for atom labels), with a change of color in every 2.5 kJ/mol and contour lines in every 10 kJ/mol (a) methanol attack on unfolded “UF” cation radical **2** conformation from QM/MM simulations with two methanol molecules in the QM region; (b) methanol attack on unfolded “UF” cation radical **2** conformation from QM/MM simulations with one methanol molecules in QM region; (C) Schematic depiction of MeOH interactions for the different intermediates; (d) methanol attack on folded “F” cation radical **2** conformation from QM/MM simulations with two methanol molecules in QM region; the energy scale of d) has been set 25 kJ/mol lower than the energy scale of a) and b), corresponding to the free energy difference of the gas-phase folded “F” and unfolded “UF” conformations from Figure 5a.

In Figure 8a, we show the 2D reaction free energy profile for methanol trapping of the tetramethoxyhexenol cation radical substrate **2**. The QM/MM simulations were performed by assuming the postulated third-order kinetics of eq 2, such that two methanol molecules participate in the cation radical trapping reaction. Correspondingly, the “QM” region consisted of the cation radical substrate **2** in addition to the two closest methanol solvent molecules, and the remainder of methanol solvent was described at the “MM” level of theory. The reaction free energy surface in Figure 8a is plotted in terms of two coordinates, R(C_α_–O_H_) and R(C_β_–O_H_), which are the distances between the oxygen atom of methanol and the two electrophilic carbon atoms of the oxidized enol ether group. It is important to note that this reaction free energy surface was computed for the cation radical substrate **2** in the unfolded “UF” geometry (see Figure 5a); the influence of the substrate conformation on methanol attack will be discussed subsequently.

The reaction profile in Figure 8a exhibits two free energy minima at R(C_α_–O_H_) ∼ 1.5 Å/R(C_β_– O_H_) ∼ 2.4 Å, and R(C_α_–O_H_) ∼ 2.5 Å/R(C_β_–O_H_) ∼ 1.5 Å, corresponding to methanol attack and bond formation with the oxidized enol ether group. The locations of these free energy minima are very similar to the intramolecular alcohol coupling reaction (Figure 6a), the difference being that, in this case, the coordinates reflect the distance from the attacking methanol oxygen atom rather than the −OH alcohol group of the substrate. Methanol trapping at both the C_α_ and C_β_ positions is a barrierless reaction, with trapping at the C_α_ position (to form the acetal) being slightly more favorable. Methanol trapping at either position is likely irreversible (on the time scales of subsequent reaction steps), given the large ∼50 kJ/mol favorable energetics associated with bond formation at either C_α_ or C_β_ position. Note that the methanol oxygen bond formation with the C_α_ (C_β_) carbon atom forces the unpaired (radical) electron to the C_β_ (C_α_) site (Figure 8c), and in Supporting Information we discuss how the second oxidation potential (relevant to mechanism Scheme 2, Figure 2) depends on which carbon site the methanol attack occurs.

**Figure 9:**
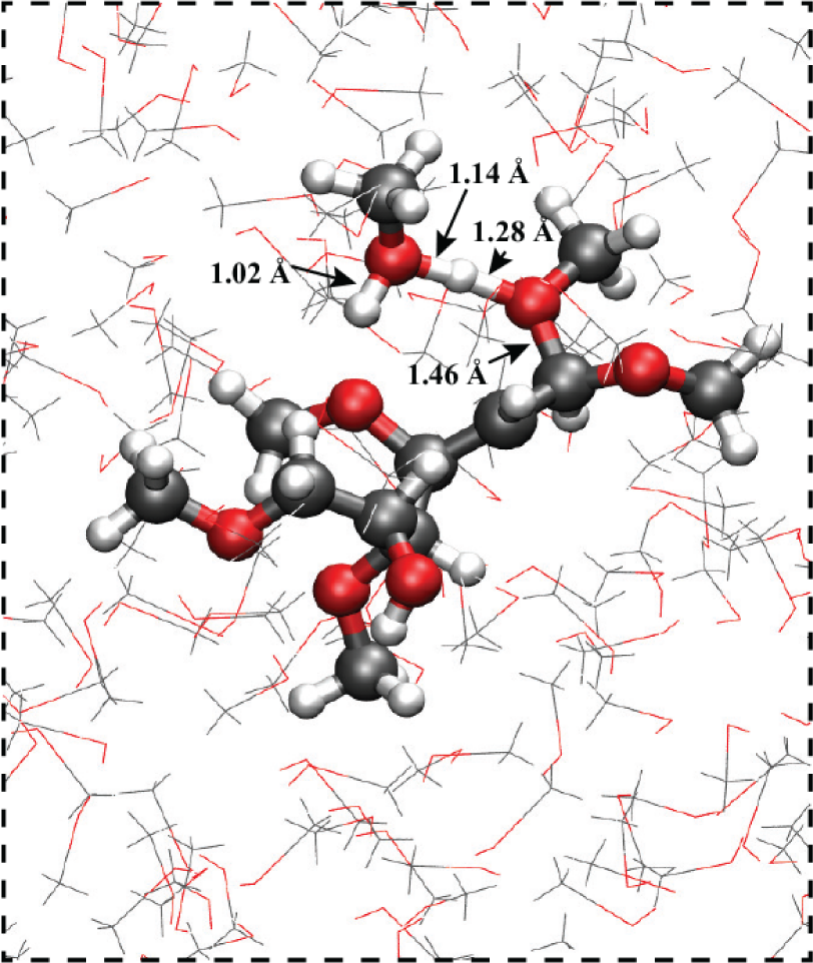
Simulation snapshot of methanol attack on the cation radical substrate **2** at the C_α_ position (in bulk methanol). The substrate and two neighboring methanol are in the QM region during the QM/MM simulation.

The second methanol solvent molecule is crucial for the nucleophilic methanol attack of cation radical **2**. The reason, similar to the intramolecular coupling reaction, is the complexation of the second methanol molecule with the acidic proton on the first methanol molecule, following nucleophilic attack. In Figure 9, we show a snapshot from the QM/MM simulation after methanol attack on the cation radical substrate **2** at the C_α_ position. The second methanol molecule forms a complex with the newly formed oxonium ion group (from the attacking methanol), with the proton shared between oxygen atoms. The O–H bond distances of the shared proton are similar to the case of intramolecular alcohol trapping (Figure 7); R(O–H) ∼ 1.14 Å for the secondary methanol molecule and R(O–H) ∼ 1.28 Å for the primary methanol molecule now covalently bonded to the C_α_ carbon atom. Similar to the geometry of the cyclic complex formed by intramolecular alcohol trapping (Figure 7), the proton sharing is asymmetric, with the shortest O–H distance for the complexing (secondary) methanol molecule.

The mechanism of secondary methanol complexation facilitating methanol attack on the enol ether cation radical is likely general to other substrates besides the tetramethoxyhexenol substrate **2** investigated here (as we will later demonstrate for methoxyoctadiene substrate **10**). However, a mechanistic detail that is specific to methanol attack at the C_α_ carbon atom of the tetramethoxyhexenol cation radical substrate **2** is a strong hydrogen bond formed between the second methanol molecule and a methoxy group on the substrate. This hydrogen bond is visually evident in the simulation snapshot shown in Figure 9. It is only observed when methanol attack occurs on the C_α_ position but not the C_β_ position, and it is likely an important reason for why attack at the C_α_ position is more thermodynamically favorable (Figure 8a) for this cation radical substrate **2**. Note that the secondary methanol molecule is a particularly strong hydrogen bond donor, sharing part of the positive charge of the shared proton. While this intermolecular hydrogen bond contributes to the stability of the methanol trapping product, it is likely not required for methanol trapping at the C_α_ position, as indicated by the computational results on the methoxyoctadiene substrate that we will present later.

To verify that complexation by the second methanol solvent molecule is crucial for the nucleophilic methanol attack to the oxidized enol ether group, we have conducted analogous QM/MM simulations of the reaction free energy profile but with the inclusion of only the primary methanol molecule and cation radical substrate **2** in the “QM” region. In this additional simulation, the secondary methanol solvent molecule is thus modeled at the “MM” level, and thus cannot covalently bond with the reactive species. The corresponding free energy profile from this additional simulation is shown in Figure 8b. The free energy profile in Figure 8b is distinctly different from the previously discussed profile in Figure 8a. In this case, there are no local free energy minima observed in the regions corresponding to the methanol trapping complexes. Complexation by the second methanol solvent molecule, as described in the QM/MM simulation of Figure 8a but not of Figure 8b, is thus crucial for the nucleophilic attack of methanol on the oxidized enol ether group. This conclusion is analogous to the case of intramolecular alcohol trapping, deduced in that case from a comparison of Figure 6a,b.

The free energy profiles computed from QM/MM simulations in Figure 8a,b strongly support the postulate of third-order kinetics (eq 2) for the nucleophilic methanol attack on the oxidized enol ether group of the substrate. As indicated by the title captions on the graphs, Figure 8a,b essentially correspond to methanol trapping reactions proceeding through overall third- order or second-order kinetics, respectively. This comparison indicates that methanol attack is favorable only with the complexation of the second methanol molecule, giving an overall third- order kinetic mechanism. As mentioned previously, kinetic studies by Oyama et al. have determined overall third-order kinetics for methanol or water trapping of anthracene-based cation radicals.^36,37^ The results demonstrated here for enol ether cation radicals **2**, namely that third-order kinetics arises from secondary methanol complexation with the newly formed oxonium ion group from the primary methanol attack, may be general for methanol trapping of other types of cation radicals, such as the anthracene molecules studied by Oyama et al.^36,37^

Another essential factor for methanol trapping of the oxidized enol ether group is the conformational geometry of the substrate. The simulation results presented and discussed so far (Figure 8a,b) were for the tetramethoxyhexenol cation radical substrate **2** in the unfolded “UF” conformation (Figure 5a). In the “UF” conformation, the −OH functional group of the substrate is relatively far from the oxidized enol ether and thus does not interfere with nucleophilic attack by methanol solvent. However, the folded “F” conformation, not the unfolded “UF” conformation, is the global free energy minima (in the gas phase, Figure 5a). As discussed, the stability of the “F” conformation is inherently due to orbital interaction between the −OH functional group of the substrate and the oxidized enol ether group; due to this orbital interaction, the oxidized enol ether is likely protected (at least to some extent) from solvent nucleophilic attack when the substrate is in the folded “F” conformation.

To investigate this conformational dependence, we conducted additional QM/MM free energy simulations for methanol trapping of the cation radical substrate **2** in the folded “F” conformation. The free energy profile for methanol trapping of the “F” conformation is shown in Figure 8d; this should be directly compared to that in Figure 8a, which is the analogous profile previously discussed for the unfolded “UF” conformation. Note that Figure 8d is computed from QM/MM simulations with the QM region composed of two methanol solvent molecules in addition to the substrate **2**, consistent with Figure 8a. From a comparison of these figures, it is clear that the folded “F” conformer (Figure 8d) is much less susceptible to methanol trapping compared to the “UF” conformer (Figure 8a). For the “F” conformer, there is only a shallow local free energy minimum for methanol trapping at the C_α_ carbon atom, and no local minimum for trapping at the C_β_ position. Due to the proximity of hydroxyl group, the C_β_ position is protected from methanol attack. Furthermore, methanol trapping at the C_α_ position exhibits an activation barrier of ∼20 kJ/mol (Figure 8d). This should be contrasted with methanol trapping of the unfolded “UF” conformer, for which trapping at both the C_α_ and C_β_ positions are highly favorable and barrierless (Figure 8a).

The overall conclusion is that there are two separate factors that promote intramolecular alcohol trapping compared to methanol trapping of the tetramethoxyhexenol cation radical substrate **2**. The first factor is the rate order difference, with intramolecular alcohol trapping being first order in methanol concentration (second order overall) and methanol trapping being second order in methanol concentration (third order overall), as verified by the free energy profiles in Figures 6 and 8. For dilute methanol conditions, which may arise either from chosen electrolyte composition and/or the modulated electrical double layer environment, this rate order difference would kinetically promote the intramolecular alcohol trapping over methanol trapping. The other important factor is the conformational energetics of the substrate. The folded “F” conformer is the most stable geometry of the substrate (Figure 5a), due to orbital interactions between the −OH group and the oxidized enol ether group (Figure S2). In the folded “F” conformation, the −OH group is already positioned in close proximity to the oxidized enol ether group, and the intramolecular trapping proceeds as a favorable and barrierless reaction upon complexation with a methanol solvent (Figure 6a). This conformation also prohibits methanol trapping (Figure 8d), since the proximal −OH group “protects” the oxidized enol ether from solvent nucleophilic attack. To allow for barrierless and favorable methanol trapping, the substrate would have to change conformations to the unfolded “UF” geometry, which is energetically unfavorable (Figure 5a).

We finally investigate methanol trapping of the methoxyoctadiene cation radical substrate **10** that is shown in Scheme 4 of Figure 3. Our study of this substrate has two purposes: first, it serves as a separate enol ether substrate to explore the generality of the conclusions previously discussed, and second, the intramolecular cyclization reaction of methoxyoctadiene (Scheme 3, Figure 3) was explored computationally in our previous work,^38^ so the present investigation of the methanol trapping side reaction (Scheme 4, Figure 3) provides a more comprehensive, computational picture of the electrosynthesis process. In the target anodic intramolecular coupling reaction of methoxyoctadiene, the enol ether cation radical **10** is a key intermediate, which is trapped by an olefin group positioned on the other side of the molecule (Scheme 3, Figure 3). The competition between intramolecular olefin trapping (Scheme 3) and methanol solvent trapping (Scheme 4) thus involves different nucleophiles (e.g., olefin and alcohol), unlike for the previously discussed tetramethoxyhexenol substrate **2** in which both intra- and intermolecular trapping involved alcohol nucleophiles. In prior computational work, we have found that the driving force for intramolecular cyclization of methoxyoctadiene cation radical substrate **10** is only moderate (and with an activation barrier), but may be enhanced by additional electrostatic stabilization from surrounding ions.^38^ Because of this, the methanol trapping side reaction (Scheme 4, Figure 3) is an important consideration for the overall electrosynthesis yield.

**Figure 10:**
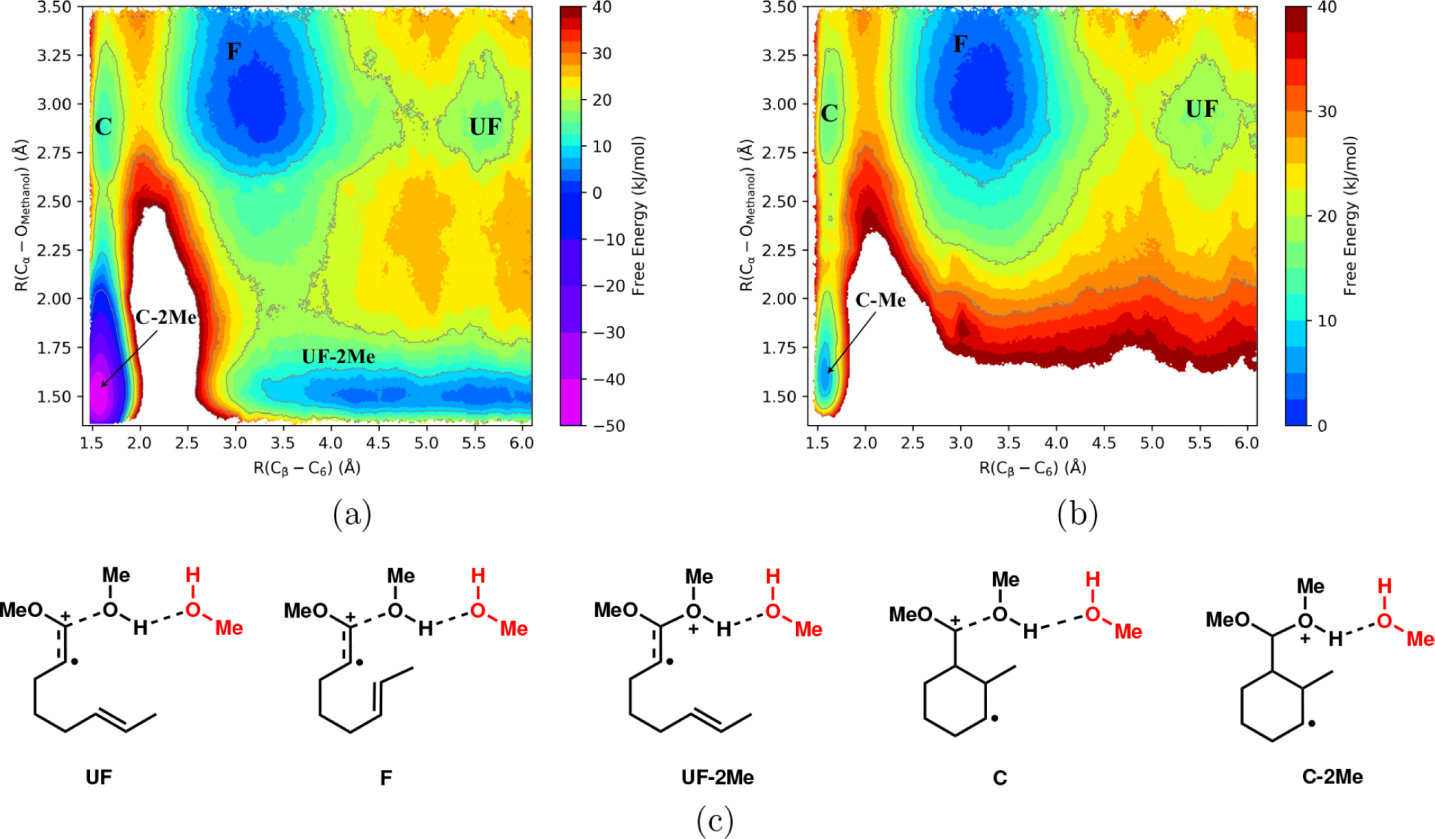
2D free energy surfaces for cyclization and methanol attack to the methoxyoctadiene cation radical substrate **10**. Change of color in every 2.5 kJ/mol and contour lines in every 10 kJ/mol (a) QM/MM simulations with two methanol molecules in the QM region. (b) QM/MM simulations with one methanol molecule in the QM region. (c) Schematic depiction of MeOH interactions for different conformations. The notation “–2Me” denotes whether a covalent bond of R(O_Methanol_-C_α_)∼ 1.5 Å has been formed with the attacking methanol molecule at a given conformation, e.g., “C-2Me” has methanol bond formation and “C” does not have a methanol bond formation.

The computational details are very similar to those previously discussed for studying the methanol trapping reaction of tetramethoxyhexenol cation radical substrate **2**. DFT-based, QM/MM simulations of the methoxyoctadiene cation radical substrate **10** were conducted in bulk methanol, with umbrella sampling used to construct reaction free energy profiles. With the expectation that methanol trapping will proceed through overall third order kinetics (based on the prior results), the QM region was chosen to consist of two methanol solvent molecules in addition to the cation radical substrate **10**. The computed 2D free energy profile for methanol trapping of methoxyoctadiene cation radical substrate **10** is shown in Figure 10a. The coordinate on the *x*-axis corresponds to the distance R(C_β_-C_6_), which dictates the intramolecular coupling of the olefin and the oxidized enol ether group, to form the 6-membered cyclized structure (**11**). While formation of a 5-membered ring is also possible,^38^ we only consider the 6-membered ring cyclized structure (**11**) here. The coordinate on the *y*-axis of Figure 10a corresponds to the distance R(O_Methanol_-C_α_), dictating methanol attack to the oxidized enol ether at the C_α_ position.

Figure 10a depicts numerous local free energy minima of methoxyoctadiene cation radical substrate **10**, which are interpreted as follows. In the top half of the free energy profile, the methanol molecule is uncoordinated (e.g., R(O_Methanol_-C_α_) ≥ 2.5 Å), and there are three local minima labeled “C”, “F”, and “UF” directly corresponding to the “cyclized”, “folded”, and “unfolded” conformations of the cation radical substrate **10** shown and discussed in Figure 5b. These three local minima in the top half of the free energy profile have similar relative stability as in the gas phase (Figure 5b); namely, the folded “F” conformation is the most stable, being ∼15 kJ/mol lower in free energy than either the cyclized “C” or unfolded “UF” conformations, in lieu of methanol complexation (for comparison, the stability of “F” is ∼20–25 kJ/mol in the gas phase, Figure 5b). The bottom half of Figure 10a depicts how the free energy of these conformations is altered upon methanol attack/complexation. Methanol complexation/bond formation starts to occur at distances of R(O_Methanol_-C_α_) ≤ 2.0 Å, which correspond approximately to the bottom third of the free energy graph. It is evident that methanol complexation leads to pronounced stabilization of the cyclized conformer and unfolded conformer; these correspond to the free energy minima labeled as “C-2Me” and “UF-2Me” at the bottom of Figure 10a.

**Figure 11:**
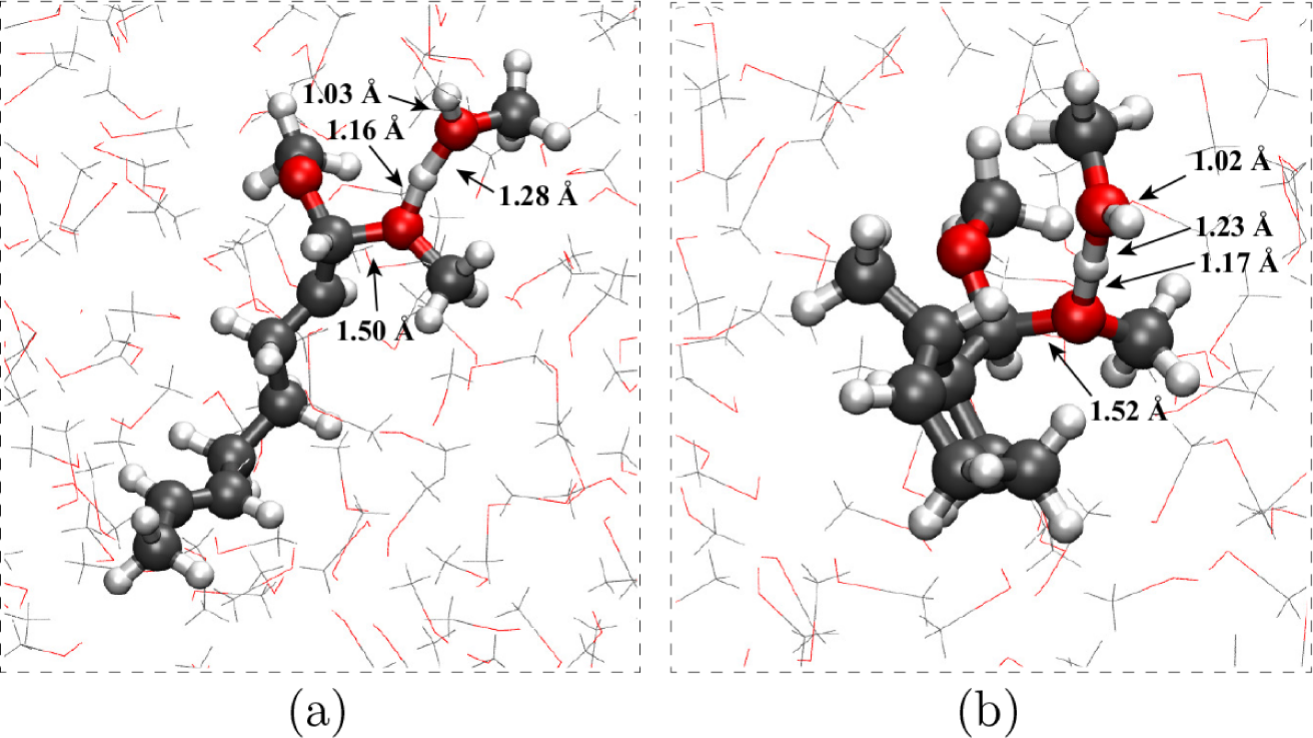
Simulation snapshots of a) unfolded (**10**) and b) cyclized (**11**) methoxyoctadiene cation radical configurations after solvent attack. The simulation snapshots are from QM/MM simulations with the substrate and two neighboring methanol molecules in the QM region.

Methanol trapping of methoxyoctadiene cation radical substrate **10** corresponds to the local free energy minimum (blue) at the bottom of Figure 10a that is labeled “UF-2Me”. Here, the distance R(O_Methanol_-C_α_) ∼ 1.5 Å indicates bond formation between methanol oxygen and the C_α_ carbon atom, and the minimum broadly extends over a large range R(C_β_-C_6_) ∼ 3.5–6.0 Å of the intramolecular carbon–carbon distance coordinate. There is a small activation barrier of ∼8.5 kJ/mol (yellow region) for methanol trapping from the “UF” to “UF-2Me” local minima (for comparison, in Figure 8, methanol trapping was barrierless). The methanol trapping depends on the complexation of a second methanol molecule to stabilize the acidic proton of the newly formed oxonium ion group. In Figure 11a, we show a simulation snapshot depicting the “UF-2Me” conformation. The complexation with a secondary methanol molecule is clearly evident. The secondary methanol shares the proton with the oxonium ion group, with O–H bond distances of 1.16 and 1.28 Å, with the longer distance for the secondary methanol oxygen atom. This is very similar to the shared proton motifs previously observed (Figures 4, 7 and 9), but with slightly different asymmetry for the shared proton O–H bond distances. To verify that complexation from a secondary methanol molecule is essential for the primary methanol attack, we conduct additional QM/MM simulations but with the QM region composed of only one methanol molecule in addition to cation radical substrate **10**. Thus, the secondary methanol molecule is treated at the “MM” level and cannot form the covalent, shared proton complex with the oxonium ion group. The free energy surface computed from this additional QM/MM simulation is shown in Figure 10b. While the upper half of the profile is nearly identical to the previously discussed free energy surface in Figure 10a, in the bottom half of the profile the minima “UF-Me” is no longer present and “C-Me” is dramatically reduced in stability. Thus, secondary methanol complexation is essential for methanol attack to the cation radical **10** to form the “UF-2Me” structure, implying third-order kinetics and fully consistent with prior analysis of the tetramethoxyhexenol cation radical substrate **2** (Figure 8).

While the intramolecular cyclization reaction of the methoxyoctadiene substrate **10** (Scheme 3, Figure 3) is not the major focus of this work, the free energy profile in Figure 10a provides important, complementary insight into this process. As evident in Figure 10a, the cyclized cation radical intermediate (**11**) is much more stable by ∼65 kJ/mol when complexed with methanol “C-2Me” relative to the same structure “C”, but not complexed to methanol. Figure 11b shows a simulation snapshot depicting the “C-2Me” structure. This structure is formed from olefin trapping of the oxidized enol ether **10** to form a 6-membered ring (**11**), followed by methanol attack at the (cationic) C_α_ position to form an acetal at C_α_. The oxonium ion formed by methanol attack then complexes to a secondary methanol, with shared proton and O–H distances of 1.17 and 1.23 Å, similar to the previous motif (Figure 11a). Formation of the “C-Me” structure (**11**) is largely dependent on complexation with the secondary methanol molecule; comparison of Figure 10a,b indicates that without complexation of the secondary methanol molecules (Figure 10b), the stability of the “C-Me” structure is reduced by ∼53 kJ/mol and is only a very shallow local minimum.

This dramatic stabilization of the “C-2Me” cyclized cation radical intermediate **11** (6-membered ring) upon methanol attack and acetal formation, likely plays an important role in the successful electrosynthesis reaction of Scheme 3 (Figure 3).^13,26^ While our previous computational work examined this intramolecular cyclization reaction for the methoxyoctadiene cation radical **10**, in that case methanol was modeled at the “MM” level only and so this complexation effect was missed.^38^ Without methanol attack and acetal formation, the 6-membered ring cyclic structure of the cation radical **11** exhibited only low-to-moderate stability relative to the folded (uncyclized) “F” conformation.^38^ In this prior work, it was observed that the electrostatic environment of the electrical double layer played an important role in enhancing the stability/formation of the 6-membered ring cyclic structure of the cation radical.^38^Figure 10a clearly indicates that methanol attack and acetal formation at the (cationic) C_α_ position is an alternative mechanism for promoting the stability of the cyclic cation radical **11** (“C-2Me” minimum), a key intermediate in the electrosynthesis process (Scheme 3, Figure 3).

**Figure 12:**
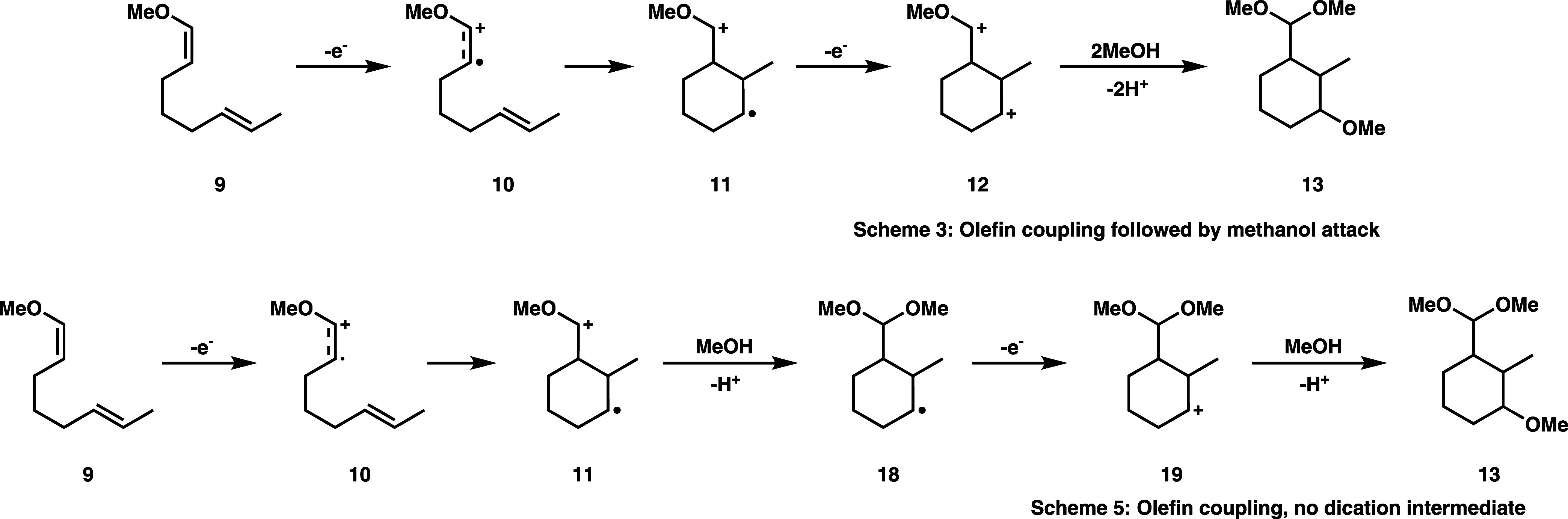
Reaction diagrams for anodic, intramolecular coupling of methoxyoctadiene substrate showing Scheme 3 mechanism of Figure 3 and proposed mechanism, which avoids the dication intermediate denoted as Scheme 5.

In Figure 12, we show a proposed reaction mechanism for the anodic, intramolecular coupling reaction of methoxyoctadiene in Scheme 5, which is based on the free energy profile in Figure 10a and avoids the dication intermediate **12** that was postulated in Scheme 3. In Scheme 5, the suggested mechanism is that, following formation of the (unstable) cyclized “C” cation radical intermediate **11**, methanol trapping at the C_α_ position to form the acetal **18** (e.g., “C-2Me”) proceeds before the second oxidation step. This “C” to “C-2Me” methanol attack reaction is highly exergonic, with a small activation barrier of ∼8 kJ/mol, with a rate that is second order in methanol concentration (Figure 10a). It is also possible that the “C” to “C-Me” methanol attack occurs through a mechanism/rate that is first order in methanol concentration (Figure 10b), but in that case, the activation barrier would be higher and the “C-Me” complex less stable. Once the “C-Me” cation radical complex is formed, it is likely that deprotonation is rapid, going to cyclic radical intermediate **18**, followed by the second oxidation and final methanol trapping steps. Whether the reaction proceeds through the dication intermediate (Scheme 3) or avoids the dication intermediate (Scheme 5) likely depends on several factors, including the rate of the second electron transfer,^13^ the methanol content of the electrolyte, and possible additional influence of the electrical double layer.^38^ We note that there has been substantial work by the Moeller group to unravel the intricate mechanisms of anodic, intramolecular coupling reactions,^9,12,13,28−30^ and our computational results simply contribute one piece to the overall mechanistic understanding of these reactions.

## 4 Conclusion

We have computationally investigated the catalytic role of methanol in nucleophilic trapping of enol ether cation radicals, focusing on two substrates that were the target of anodic, intramolecular coupling reactions, as previously conducted by the Moeller group. Empirically it had been observed that the yield of these and similar electrosynthesis reactions was substantially modulated by the nature of the electrolyte, and in particular its methanol content.^9,13,28−30^ Upon alcohol trapping of an enol ether cation radical, which is a key step in the intramolecular cyclization of the tetramethoxyhexenol substrate **2**, the acidic proton of the newly formed oxonium ion group forms a shared proton complex with a methanol molecule; this motif is reminiscent of the well-known Zundel structure of . This methanol complexation is essential for the alcohol trapping to occur, stabilizing the cyclic structure by ∼60 kJ/mol (relative to no methanol) with the intramolecular alcohol trapping proceeding in a barrierless manner. The conclusion is that intramolecular alcohol coupling of enol ether cation radicals proceeds through second order kinetics with a first order dependence on methanol concentration. It is important to note that our prediction of a *barrierless* intramolecular alcohol trapping step is consistent with the “Nernstian shift” concept discussed by both Little and Moeller, in which a rapid cyclization step will shift the first oxidation potential of the substrate to a less positive (easier to oxidize) value relative to oxidation of the bare functional group.^31,66^

For anodic electrosynthesis reactions in methanol-based electrolytes, potential side reactions involving the methanol trapping of cation radical intermediates are well-known. We have computed free energies for methanol trapping of the enol ether cation radical substrates, and indeed observe that methanol trapping is barrierless (tetramethoxyhexenol substrate **2**) or with small ∼8.5 kJ/mol barrier (methoxyoctadiene substrate **10**), for certain substrate conformations. However, analogous to intramolecular alcohol trapping, methanol trapping relies on complexation with a secondary methanol molecule so that a shared-proton complex is formed with the acidic proton of the oxonium ion group. Thus, methanol trapping of enol ether cation radicals proceeds through third order kinetics, with a second order dependence on the methanol concentration. One would expect these conclusions to generalize to other protic, nucleophilic solvents such as water or other alcohols and other types of cation radicals in addition to oxidized enol ethers. Indeed, prior kinetic studies of Oyama et al.^36,37^ concluded that methanol or water trapping of anthracene derived cation radicals proceeds through similar third order kinetics, suggesting generalization of the trapping mechanism to other types of cation radicals. The unequivocal conclusion from our free energy computations suggest revised rate constants for prior work on methanol trapping of enol ether cation radicals, that previously assumed overall second order kinetics.,^32,34,35^

The success of anodic intramolecular coupling reactions,^9,13,28−30^ as competing with methanol trapping side reactions, may often benefit from the stability of a precyclized, “folded” cation radical conformation, with orbital interaction between the nucleophile and oxidized enol ether group. Indeed such “folded” conformations exhibit additional stability of ∼20–25 kJ/mol compared to “unfolded” conformations, for the tetramethoxyhexenol and methoxyoctadiene cation radical substrates, despite electrophile/nucleophile distances being significantly longer than covalent bonds (e.g., R(C–C) ∼ 3.3 Å, R(C–O) ∼ 2.2 Å). In the folded conformation, the oxidized enol ether group is largely protected from methanol attack, given the intramolecular nucleophile interaction. For barrierless or low-barrier (∼8.5 kJ/mol) methanol trapping to occur, the radical cation would have to undergo a conformational transition from the “folded” to “unfolded” geometry, with associated barrier of ∼20–30 kJ/mol depending on substrate and solvent (i.e our computations show this barrier is lower in bulk methanol compared to gas-phase). Thus, if intramolecular nucleophilic coupling proceeds with barriers lower than ∼20–30 kJ/mol, it would be kinetically favored over solvent methanol trapping. This is in addition to the rate order difference for the processes, whereby intramolecular trapping would be favored over methanol trapping at low methanol content due to the respective first and second order rate dependence on methanol concentration.

Consistent with empirical observations of Moeller and coworkers,^9,13,28−30^ our results highlight why the yield of anodic intramolecular coupling reactions may often be highly sensitive to the complex nature of the electrical double layer at the anode. The affinity for the substrate to approach/contact the anode surface, required for the initial oxidation, will depend on the solubility of the substrate within the highly charged, electrical double layer environment.^29^ Furthermore, barrierless or low-barrier reactions such as the alcohol trapping of enol ether cation radicals investigated here are expected to occur very rapidly before the oxidized substrate can diffuse out of the double layer back to the bulk electrolyte environment. In these cases the nucleophilic trapping of cation radical intermediates may be significantly modulated within the double layer due to the large electric fields and high ionic concentrations expected at the high working potentials (e.g., 1–1.5 V vs Ag/AgCl) utilized in the electrolysis. Furthermore, the different (third vs second) rate orders of intramolecular alcohol trapping and methanol trapping will lead to pronounced differences in overall reaction rates for different double layer concentrations. In this regard, computational approaches may aid experimental design of optimal electrosynthesis conditions, though characterization of concentration profiles and reaction free energies within the double layer at working electrolysis conditions.^38^

## Data Availability

The data underlying this study are available in the published article and its Supporting Information

## Supporting Information Available

simulation_files (ZIP)
DFT functional and basis set benchmarks compared to MP2/cc-pVTZ level of theory; optimized geometries and parametrized charges for +1 oxidation state of the 1,3,4,6- tetramethoxyhex-5-en-2-ol substrate; relative energy of the “unfolded” and “folded” conformation of the 1,3,4,6-tetramethoxyhex-5-en-2-ol substrate with respect to intrinsic reaction coordinates; characterization of the oxonium complexes; first and second oxidation potentials of 1,3,4,6-tetramethoxyhex-5-en-2-ol substrate (methanol coordinated) (PDF)
