# Supplementary material for: Catalytic Role of Methanol in Anodic Coupling Reactions Involving Alcohol Trapping of Cation Radicals
Source: J Org Chem. 2024 Dec 3;89(24):18353–69. doi: 10.1021/acs.joc.4c02227 (PMC11667722; doi:10.1021/acs.joc.4c02227)
Supplement: Supplementary file 2 — jo4c02227_si_002.pdf [file jo4c02227_si_002.pdf]

Supporting Information for “Catalytic Role of Methanol in Anodic Coupling Reactions involving Alcohol Trapping of Cation Radicals”

Shahriar N. Khan, John H. Hymel, John P. Pederson, and Jesse G. McDaniel*

School of Chemistry and Biochemistry, Georgia Institute of Technology, Atlanta, Georgia 30332-0400, United States

E-mail: mcdaniel@gatech.edu

Contents

S1 DFT Functional and Basis Set Benchmarking with respect to MP2/cc-pVTZ Level of Theory **S2**

S2 Optimized Geometries and Fit Charges for +1 Oxidation State of the 1,3,4,6- tetramethoxyhex-5-en-2-ol substrate **S5**

S3 Relative Energy of the ‘unfolded’ and ‘folded’ conformation of the 1,3,4,6-tetramethoxyhex-5-en-2-ol substrate with respect to Intrinsic Reaction Coordinates **S7**

S4 Characterization of the oxonium complexes: A preparatory step for deprotonation **S9**

S5 First and Second Oxidation Potentials of 1,3,4,6- tetramethoxyhex-5-en-2-

ol Substrate (Methanol Coordinated) Computed in Implicit Solvent (Methanol) S10

References S12

S1 DFT Functional and Basis Set Benchmarking with respect to MP2/cc-pVTZ Level of Theory

Considering the limitations of DFT for describing the electronic structure of cation radicals, and in particular two-center, three-electron bonding of such substrates,[1,2] we have benchmarked the accuracy of the PBE0/6-31G and PBE0/6-31G* levels of theory with respect to MP2/cc-pVTZ level of theory. For computational tractability of the MP2 benchmarks, a simplified enol ether substrate was considered in which the three methoxy groups were removed from the scaffold of the 1,3,4,6-tetramethoxyhex-5-en-2-ol substrate, as shown in Figure S1a. This cation radical substrate is complexed with one methanol molecule, as necessary for the intramolecular cyclization reaction. Geometry optimization of the reactants and products was done independently with the three methods, MP2/cc-pVTZ, PBE0/6-31G, PBE0/6-31G*, with implicit solvent (methanol) and D3 dispersion correction for DFT methods. Optimized geometries were found for the reactants and products except for the folded reactant at PBE0/6-31G level of theory; for the folded reactant at PBE0/6-31G level, we thus do a constrained geometry optimization in which the C-O distance reaction coordinate is held fixed at the optimized distance from the PBE0/6-31G* optimization. The reaction energies going from the folded, uncyclized cation radical to the cyclized cation radical, as predicted by the different levels of theory are given in Figure S1a. For this comparison, we set the energy reference of the cyclized cation radical "product" to be the same for all methods, as the methods likely have a larger disparity in the electronic structure description of the folded, uncyclized cation radical structure. This is because the folded, uncyclized cation

radical structure is likely described by a two-center, three-electron bond between the oxygen nucleophile (lone pair) and cation radical, for which DFT is known to exhibit systematic error.

Figure S1a shows that reaction energy predictions from PBE0/6-31G and PBE0/6-31G* "bound" the MP2/cc-pVTZ prediction, with deviation of either DFT approach being $\sim$ 7-9 kJ/mol from the MP2/cc-pVTZ benchmark result. Of the DFT calculations, the PBE0/6-31G* calculation has the "better" 6-31G* basis set (including polarization functions), and in this case PBE0/6-31G* overstabilizes the folded, uncyclized cation radical structure, due to the tendency to overstabilize two-center, three-electron bonds.[1,2] In contrast, the smaller basis set utilized in PBE0/6-31G, tends to understabilize the folded, uncyclized cation radical structure (two-center, three-electron). This error cancellation means that both PBE0/6-31G* and PBE0/6-31G exhibit essentially equivalent predictive accuracy compared to the MP2/cc-pVTZ benchmark, albeit predicting opposite stability trends; i.e. folded, uncyclized cation radical structure is artificially stabilized (de-stabilized) by PBE0/6-31G* (PBE0/6-31G).

In Figure S1b, we show an additional benchmark of the PBE0/6-31G and PBE0/6-31G* methods for the 1,3,4,6-tetramethoxyhex-5-en-2-ol cation radical substrate that is the subject of this work. Because of the size of this substrate it was intractable to do the similar MP2/cc-pVTZ geometry optimization. The reaction and product structures were optimized as described above, for the previous enol ether cation radical substrate. From Figure S1b, it is seen that the discrepancy in predictions of reaction energy between the PBE0/6-31G and PBE0/6-31G* methods is $\sim$ 10 kJ/mol for the 1,3,4,6-tetramethoxyhex-5-en-2-ol cation radical substrate, as compared to the larger discrepancy of $\sim$ 16.5 kJ/mol for the enol ether substrate in Figure S1a. This suggests that the benchmarks in Figure S1a may provide an upper bound estimate for the error of the PBE0/6-31G method as applied to the substrates investigated in this work.

Table S1 presents additional benchmarking for DFT functionals B3LYP and M06-2X.

The reaction energies of the simplified enol ether is computed at B3LYP-D3/6-31G, B3LYP-D3/6-31G*, M06-2X-D3/6-31G, and M06-2X-D3/6-31G* level of theory. To be consistent with figure S1, the reaction energy is defined as $E_{Reactant}$ - $E_{Product}$. In comparison to MP2/cc-pVTZ, the B3LYP functional overstabilizes the two-center three-electron folded structure. On the other hand, M06-2X functional is very sensitive to the choice of basis sets and exhibits larger deviations with respect to MP2/cc-pVTZ.

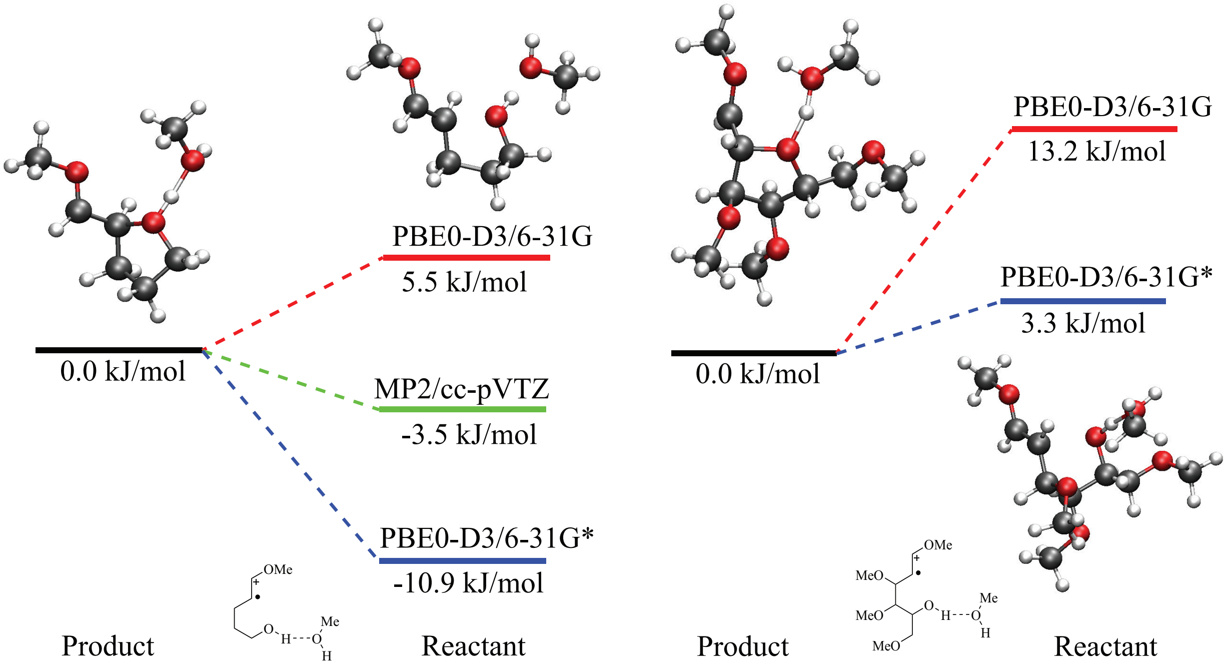

(a) (b)

Figure S1: (a) Reaction energies of a simplified enol ether comparing PBE0/6-31G and PBE0/6-31G* with respect to MP2/cc-pVTZ level of theory. (b) Reaction energies of 1,3,4,6-tetramethoxyhex-5-en-2-ol substrate comparing PBE0/6-31G and PBE0/6-31G* basis sets.

Table S1: Reaction energy in kJ/mol for the simplified enol ether (FigureS1a). The reaction energy is defined as $E_{Reactant}$ - $E_{Product}$

Method	6-31G	6-31G*	cc-pVTZ
PBE0-D3	5.5[a]	-10.9	
B3LYP-D3	-10.0	-29.0[b]	
M06-2X-D3	22.5[a]	-17.2[a]	
MP2			-3.5

[a] Constrained Geometry Optimization for the Reactant.
[b] Constrained Geometry Optimization for the Product.

S2 Optimized Geometries and Fit Charges for +1 Oxidation State of the 1,3,4,6- tetramethoxyhex-5-en-2-ol substrate

In the QM/MM-MD simulations, for the free energy calculations of alcohol trapping of +1 oxidized 1,3,4,6- tetramethoxyhex-5-en-2-ol (cation radical) substrate, charge fitting method, distributed multi-pole expansion analysis (DMA) was employed as described in the main text. Optimized geometries at PBE0/6-31G level and fitted charges are given in Table S2 for the oxidized substrate. The oxidized substrate has an energetic preference for the 'folded' geometry (*vide infra*). So, for charge fitting of the +1 state, optimized geometry of the 'folded' configuration was used.

Table S2: Optimized Geometry and Fit Charges for +1 Oxidation State of the 1,3,4,6-tetramethoxyhex-5-en-2-ol Substrate

Atom	X	Y	Z	Charge(Radical Cation)
C	1.486	1.462	−4.342	0.1491
O	1.515	1.621	−2.878	−0.3597
C	0.436	1.362	−2.155	0.2623
C	0.519	1.523	−0.778	0.0894
C	−0.634	1.385	0.144	0.2328
H	−1.518	1.922	−0.246	0.0413
O	−0.202	1.935	1.385	−0.5364
C	−1.229	2.452	2.278	0.2048
C	−1.030	−0.128	0.299	0.3679
H	−1.326	−0.521	−0.686	−0.0487
O	−2.066	−0.308	1.251	−0.5783
C	−3.421	−0.157	0.757	0.1973
C	0.202	−0.857	0.789	0.2370
H	0.510	−0.445	1.755	0.0503
O	1.190	−0.540	−0.221	−0.6536
C	0.087	−2.363	0.880	0.3517
O	1.445	−2.785	1.106	−0.6501
C	1.609	−4.201	1.344	0.2470
H	2.482	1.742	−4.673	0.1239
H	0.735	2.133	−4.764	0.0714
H	1.273	0.420	−4.587	0.0715
H	−0.460	1.020	−2.667	0.0852
H	1.429	1.934	−0.355	0.1565
H	−0.685	2.976	3.062	0.0852
H	−1.821	1.640	2.703	0.0467
H	−1.876	3.162	1.748	0.0038
H	−4.070	−0.434	1.585	0.1048
H	−3.602	−0.824	−0.093	0.0262
H	−3.630	0.879	0.466	0.0178
H	2.038	−0.981	−0.004	0.5129
H	−0.301	−2.791	−0.055	−0.0257
H	−0.574	−2.646	1.708	0.0101
H	2.674	−4.360	1.507	0.0774
H	1.276	−4.787	0.479	0.0102
H	1.051	−4.513	2.235	0.0178

S3 Relative Energy of the 'unfolded' and 'folded' conformation of the 1,3,4,6- tetramethoxyhex-5-en-2-ol substrate with respect to Intrinsic Reaction Coordinates

Here we present more insights on the electronic structure of the 1,3,4,6- tetramethoxyhex-5-en-2-ol substrate. This cation radical substrate has two important conformations those have further consequences on the reaction mechanism. We have dubbed them as "folded" (F) and "unfolded" (UF) conformations where the former represents a quantum mechanical interaction (i.e. two center, three electron bond) between the lone-pair of O_H and the positive dipole of the cation radical. At first, gas phase (with implicit solvent methanol and D3 dispersion) geometry optimization has been done followed by an Intrinsic Reaction Coordinate (IRC) calculation utilizing Nudged Elastic Band (NEB) method. Figure S2 presents the relative electronic energies of the conformers. The "folded" and "unfolded" substrates, at their optimized geometries, have C_β-O_H distance 2.2 Å and 4.2 Å respectively. The "folded" conformation is energetically (electronic energy) more stable than the "unfolded" geometry by 25 kJ/mol. Based on the IRC, the transformation from "unfolded" to "folded" geometry has an energy barrier of 14 kJ/mol (Figure S2). Energetic stability of the "folded" geometry is mostly quantum mechanical in nature, likely described as a two-center, three electron bond between C_β-O_H sites (2.2 Å). Figure S2c) depicts the iso-surface of the HOMO, orbital overlap between cation radical and O_H.

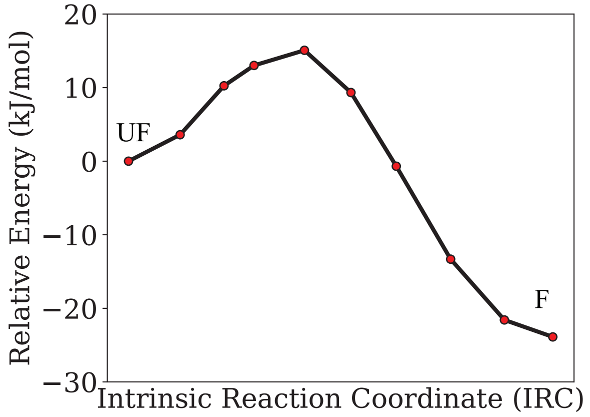

(a)

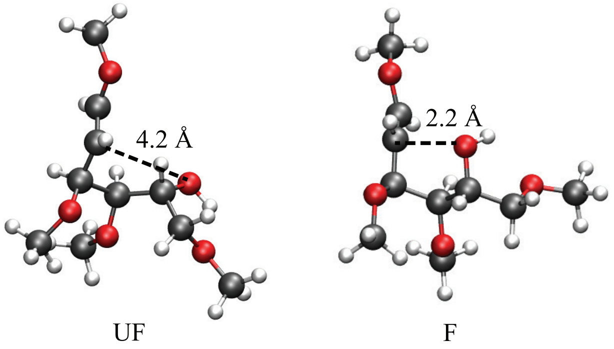

(b)

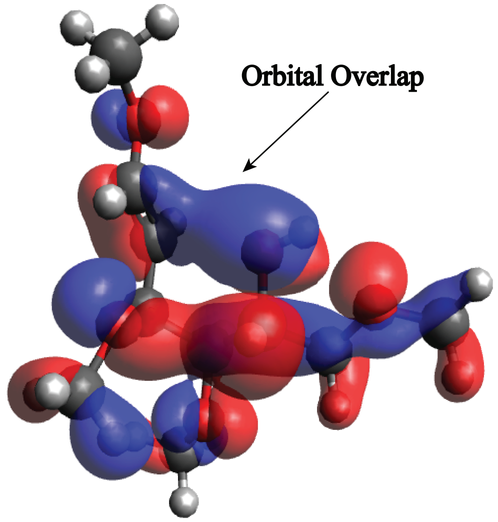

(c)

Figure S2: (a) Relative electronic energy of the “folded” (F) and “unfolded” (UF) conformation of the cation radical following the Intrinsic Reaction Coordinate (IRC). (b) Optimized geometries of the “folded” and “unfolded” conformation at PBE0/6-31G level of theory. (c) Orbital iso-surface (HOMO) of the “folded” conformation depicting the interaction of -O_H and cation radical.

S4 Characterization of the oxonium complexes: A preparatory step for deprotonation

Characterization of the oxonium complexes is done by analysing the O-H bond distance of the substrate considering one catalytic methanol in the quantum mechanical description from 12 ps QM/MM simulation. Figure S3 represents the distribution of O-H bond distances for the cyclic substrate. For the cyclic product, the range of O-H bond distances is distributed between 1.0 to 1.6 Å where the majority of the distribution falls within 1.2 to 1.4 Å. This elongated range of O-H bond distance is typical for the formation of the oxonium complexes. For comparison, the O_{sol}-H distances (red) in figure S3 are centered around 1.1 Å which indicates the formation of oxonium ion with the solvent methanol. The final cyclic product involves a deprotonation step, and the complex formed by the catalytic methanol likely serves as the initialization of deprotonation. In our simulations, we have not observed complete deprotonation of the substrate within the simulation time length, suggesting an (free) energetic barrier to deprotonation; however, this deprotonation step has not been the focus/or studied thoroughly.

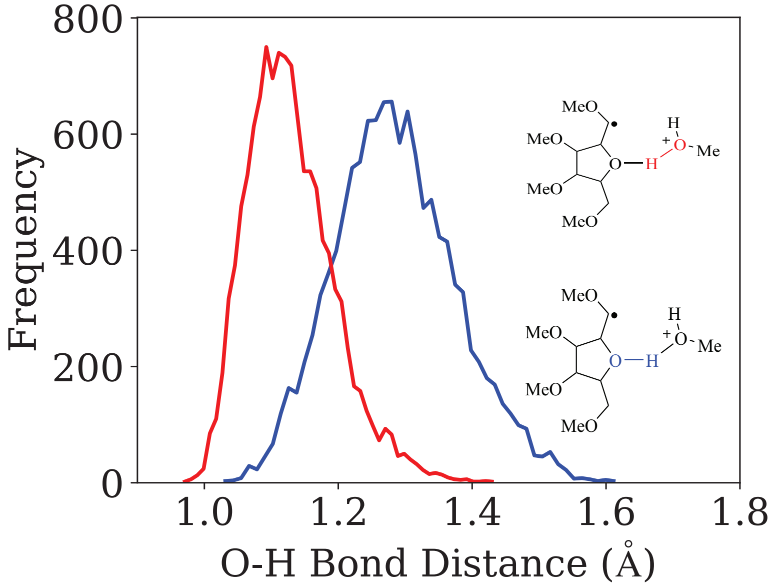

Figure S3: Distribution of O-H bond distances for the cyclized substrate.

S5 First and Second Oxidation Potentials of 1,3,4,6-tetramethoxyhex-5-en-2-ol Substrate (Methanol Co-ordinated) Computed in Implicit Solvent (Methanol)

Table S3: Oxidation Potentials of 1,3,4,6- tetramethoxyhex-5-en-2-ol Substrate (Methanol coordinated)

Species Figure S4	1st Ox./ 2nd Ox.	Absolute Oxidation Potential (eV)	Oxidation Potential (eV) vs Ag/AgCl
a	1st	5.43	0.83
b	2nd	5.40	0.80
c	2nd	5.80	1.20
d	2nd	6.80	2.20
e	2nd	7.24	2.64

For the calculation of the 1st and 2nd oxidation potentials, we have calculated the ionization energies of the substrate with one/two methanol coordination. Vertical ionization energies have been calculated utilizing PBE0/6-31G level of theory with implicit solvent

(methanol) and D3 dispersion corrections.

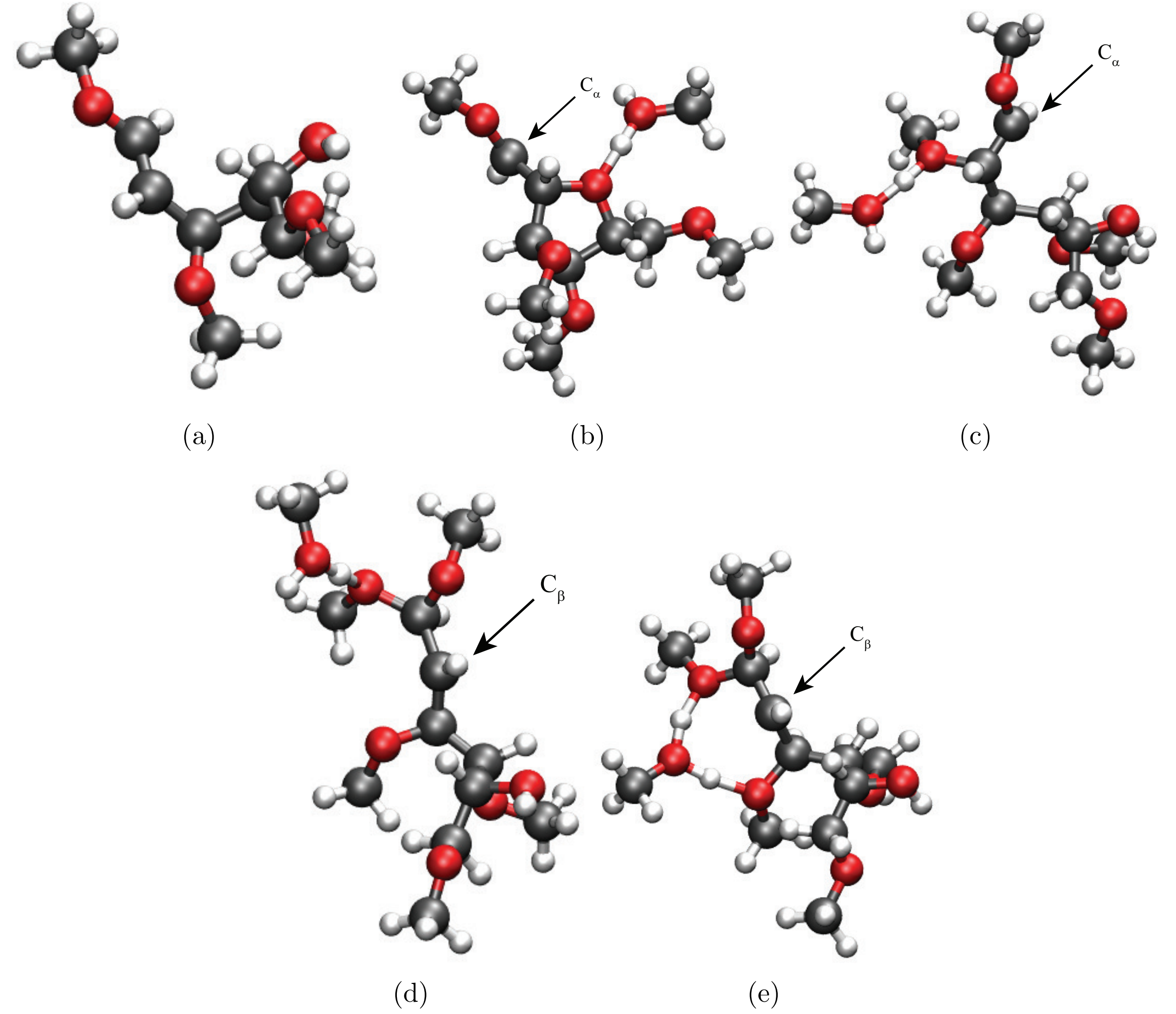

Figure S4: Representative geometric configuration of the 1,3,4,6- tetramethoxyhex-5-en-2-ol substrate for the calculated 1st and 2nd oxidation potentials. Arrow indicates the carbon site (C_α/C_β) of the 2nd oxidation. (a) Unfolded substrate (no methanol coordination), (b) Cyclized 5-membered ring product (one methanol coordination), (c) Unfolded substrate with methanol attach at C_β (two methanol coordination), (d) Unfolded substrate with methanol attach at C_α (two methanol coordination), (e) Unfolded substrate with methanol attach at C_α (two methanol coordination with intramolecular hydrogen bond)

References

(1) Sodupe, M.; Bertran, J.; Rodríguez-Santiago, L.; Baerends, E. J. Ground State of the (H2O)2+ Radical Cation: DFT versus Post-HartreeFock Methods. *The Journal of Physical Chemistry A* **1999**, *103*, 166–170.

(2) Braida, B.; Hiberty, P. C.; Savin, A. A Systematic Failing of Current Density Functionals: Overestimation of Two-Center Three-Electron Bonding Energies. *The Journal of Physical Chemistry A* **1998**, *102*, 7872–7877.
